# Dynamical modeling of miR-34a, miR-449a, and miR-16 reveals numerous DDR signaling pathways regulating senescence, autophagy, and apoptosis in HeLa cells

**DOI:** 10.1038/s41598-022-08900-y

**Published:** 2022-03-22

**Authors:** Shantanu Gupta, Pritam Kumar Panda, Ronaldo F. Hashimoto, Shailesh Kumar Samal, Suman Mishra, Suresh Kr. Verma, Yogendra Kumar Mishra, Rajeev Ahuja

**Affiliations:** 1 Instituto de Matemática e Estatística, Departamento de Ciência da Computação, Universidade de São Paulo, Rua do Matão 1010, São Paulo, SP 05508-090 Brazil; 2 Condensed Matter Theory Group, Materials Theory Division, Department of Physics and Astronomy, Uppsala University, Box 516, 751 20 Uppsala, Sweden; 3 Unit of Immunology and Chronic Disease, Institute of Environmental Medicine, Karolinska Institutet, 17177 Stockholm, Sweden; 4 School of Biotechnology, KIIT University, Bhubaneswar, 751024 India; 5 Mads Clausen Institute, NanoSYD, University of Southern Denmark, Alsion 2, 6400 Sønderborg, Denmark

**Keywords:** Dynamic networks, Gene regulatory networks, Cellular signalling networks

## Abstract

Transfection of tumor suppressor miRNAs such as miR-34a, miR-449a, and miR-16 with DNA damage can regulate apoptosis and senescence in cancer cells. miR-16 has been shown to influence autophagy in cervical cancer. However, the function of miR-34a and miR-449a in autophagy remains unknown. The functional and persistent G1/S checkpoint signaling pathways in HeLa cells via these three miRNAs, either synergistically or separately, remain a mystery. As a result, we present a synthetic Boolean network of the functional G1/S checkpoint regulation, illustrating the regulatory effects of these three miRNAs. To our knowledge, this is the first synthetic Boolean network that demonstrates the advanced role of these miRNAs in cervical cancer signaling pathways reliant on or independent of p53, such as MAPK or AMPK. We compared our estimated probability to the experimental data and found reasonable agreement. Our findings indicate that miR-34a or miR-16 may control senescence, autophagy, apoptosis, and the functional G1/S checkpoint. Additionally, miR-449a can regulate just senescence and apoptosis on an individual basis. MiR-449a can coordinate autophagy in HeLa cells in a synergistic manner with miR-16 and/or miR-34a.

## Introduction

**Figure 1:**
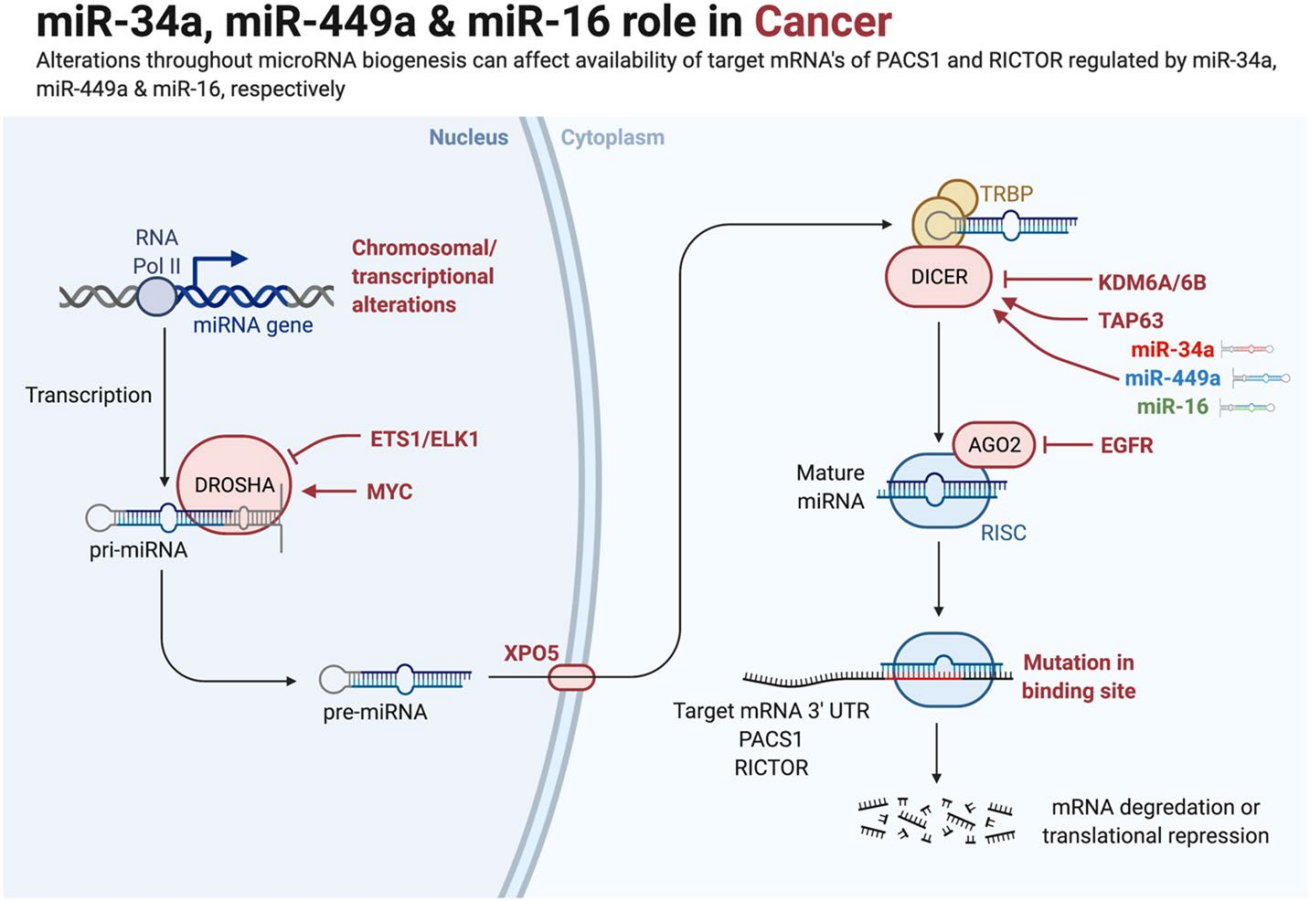
Schematic illustration depicting the role of microRNAs (miR-34a, miR-449a, and miR-16) in Cancer. Alterations throughout miRNA biogenesis can affect the availability of target mRNA of PACS1 and RICTOR regulated by miR-34a, miR-449a, and miR-16, respectively. miRNA genes are transcribed by RNA polymerase II to produce the large primary transcripts pri-miRNAs. The pre-miRNAs are processed by another RNase III enzyme Dicer to a ~ 20–22-nucleotide miRNA/miRNA* duplex. After the duplex is unwound, the mature miRNA is incorporated into a protein complex termed RISC. A miRNA-loaded RISC mediates gene silencing via mRNA cleavage and degradation on the complementarity between the miRNA and the targeted mRNA transcript. In addition, miRNAs may function as ligands to directly bind with Toll-like receptors (TLR), triggering downstream signaling pathways. Methyltransferase-like 3 (METTL3) is recently discovered to methylate pri-miRNAs, marking them for recognition and processing by DGCR to yield mature miRNA.

MicroRNAs (miRNAs) are well-characterized master regulators of gene expression that play a critical role in fundamental biological processes^1^. Recent research suggests that manipulating miRNA expression may reflect DNA damage challenges (e.g., radiation, chemotherapy, and reactive oxygen species (ROS)). Veena et al.^2^ have discovered that Phosphofurin acidic cluster sorting protein 1 (PACS1) expression is increased in HeLa cells^2^ (see Fig. 1). Additionally, they found that microRNA-34a (miR-34a) and/or microRNA-449a (miR-449a) targeting PACS1 can promote DNA damage response (DDR) in Hela cells. Indeed, overexpression of PACS1 reduces the amounts of phosphorylated histone H2AX (H2AX), hence enhancing the checkpoint's barrier.

In this scenario, miR-34a and/or miR-449a targeting PACS1 can boost H2AX production in HeLa cells. Additionally, it has been demonstrated that increased expression of Cdc25A and Wip1 leads to the advancement of HeLa cancer^3,4^. As a result, MicroRNA-16 (miR-16) is being highlighted as a potential inhibitor of Wip1/Cdc25A activity in cervical cancer^3,5^. Pothof et al.^3^ previously established that overexpression of miR-16 binds Cdc25A and inhibits the G1/S checkpoint in HeLa cells. Similarly, miR-16 targeting Cdc25A and/or Wip1 modulates H2AX expression and enhances DDR pathways in cervical cancer^3,6^.

It is widely established that all of these miRNAs (miR-34a, miR-449a, and miR-16) may control various signaling pathways involved in DNA damage-induced cell cycle arrest, senescence, and apoptosis. Despite this, current research shows that these miRNAs may potentially play a role in autophagy signaling. Huang et al.^7^ demonstrated the involvement of miR-16 in the activation of autophagy by targeting mTOR-2 within this framework. Additionally, they revealed that overexpression of miR-16 in HeLa cells inhibits G1 arrest and death^7^.

Similarly, Rathod et al.^8^ established that miR-34a targets mTOR-2 directly. When glioma and glioma stem cell lines were compared to normal brain tissue, they discovered that miR-34a expression was reduced while the AKT-mTOR pathway was elevated^8^. Additionally, they established that mTOR-2 and miR-34a had a negative connection and revealed that miR-34a was specifically targeting mTOR-2 in matched cell lines^8^. Furthermore, Torossian et al.^9^ shown that miR-34a is required for the regulation of autophagic and apoptotic cell death in lymphoma cells by directly targeting BCL2 expression^9^.

miRNAs are key participants in the autophagy process, which progresses by initiating autophagy, proceeds with developmental stages, and ends by the degradation stage that has accumulated during autophagy^10^. miRNAs have been shown to affect ATGs (autophagy-related genes) and associated regulator expression processes, including induction, vesicle nucleation, phagophore assembly, lysosomal fusion, and degradation^10^.

The PI3K-AKT-mTOR, TP53-mTOR, and Ca2+-AMPK-mTOR pathways are among the upstream nutrient and energy signals involved in autophagy induction regulation, and certain miRNAs have been shown to modify these signals to cause phagophore induction^11–13^. Under genotoxic stress, TP53 and HMGB1 form a complex that has a reciprocal inhibitory function, controlling SIRT1-mTOR signaling downstream. Via the Ca2+-AMPK-mTOR pathway, calcium-metabolizing enzymes including transient receptor potential melastatin 3 (TRPM3) and Drosophila inositol 1,4,5-triphosphate kinase 2 (IP3K2) regulate autophagy initiation^13^.

In HeLa cells, expressing a wild-type p53 protein, they are rendered deficient functional p53 due to the expression of the human papillomavirus (HPV) protein E6^14^, which accelerates p53 degradation^14^. It is an assumption that the accumulation of p53 may not control a functional G1/S checkpoint (due to HPV proteins)^15^. However, increasing evidence suggests that the p53 pathway is important and may induce the G1/S checkpoint in DDR^14,16,17^. In addition to the p53 pathway, other signaling pathways such as ATM, AMPK, and MAPK are linked in the regulation of cell fate determination (senescence, autophagy, and apoptosis) at the G1/S checkpoint in HeLa cells^17,18^.

**Figure 2:**
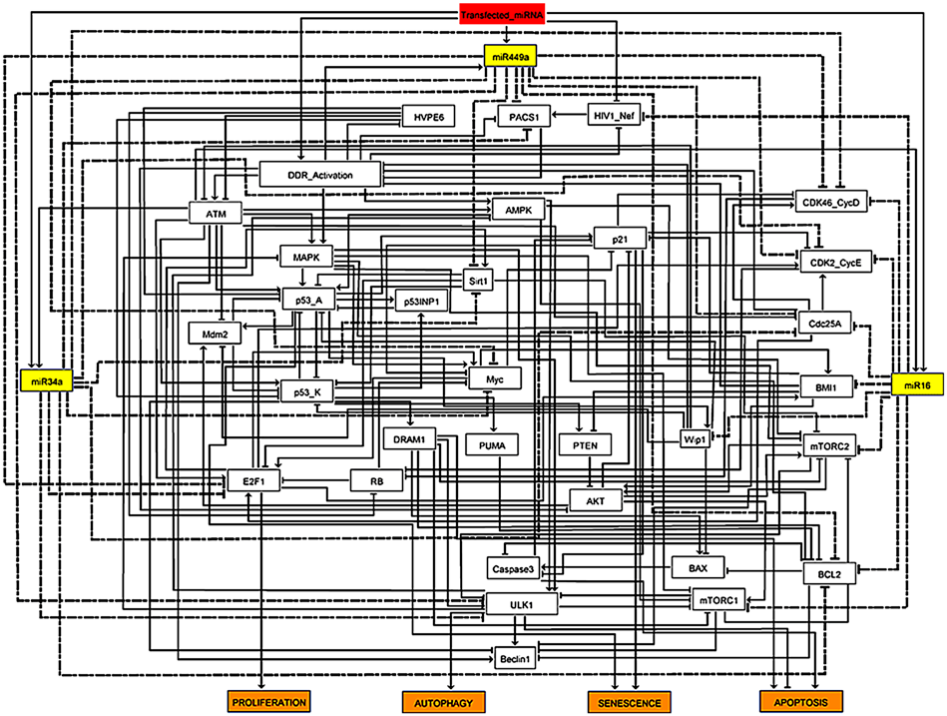
Synthetic gene regulatory network for the functional G1/S checkpoint. Arrows denote activations and hammer-head arcs represent inhibitions, respectively. Dashed hammer-head arcs represent targets of miRNA-34a, miR-449a and miR-16. The yellow rectangular nodes represent miRNAs and the red one denotes transfected miRNAs which induces DNA damage response in HeLa cells. The network outputs are in orange rectangular nodes represents proliferation or autophagy or apoptosis or senescence.

In this context, the study of Veena et al.^2^ and Huang et al.^7^ provided evidence that transcription of these miRNAs (miR-16, miR-34a, and miR-449a) are associated with the induction of autophagy, apoptosis, and senescence at the functional and stable G1/S checkpoint in HeLa cells. However, the precise molecular mechanisms of these miRNAs (miR-16, miR-34a, and miR-449a) synergistically or individually in cervical cancer are still unclear. Therefore, we present a synthetic Boolean model for the functional G1/S checkpoint in HeLa cells involving the p53, AMPK, and MAPK pathways (see Fig. 2). To our knowledge, this is the first study considering the role of three miRNAs (miR-34a, miR-449a, and miR-16) in the three major DDR signaling pathways (p53, MAPK, and AMPK) in cervical cancer.

## Results

### The network construction and its fixed points

"Transfection of miRNAs" is a single input to the model, represented by a rectangle node in red (see Fig. 2). MiRNAs are shown as yellow rectangular nodes (miR-16, miR-34a, and miR-449a). Each miRNA's target is shown by a dashed hammer-head arc, whereas arrows indicate activation and hammer-head arcs indicate inhibition, respectively. Proliferation, autophagy, apoptosis, and senescence are represented by the model outputs in orange-colored rectangular nodes. The network is composed of 32 proteins and three miRNAs, which are connected by 145 direct interactions (see Fig. 2).

**Figure 3:**
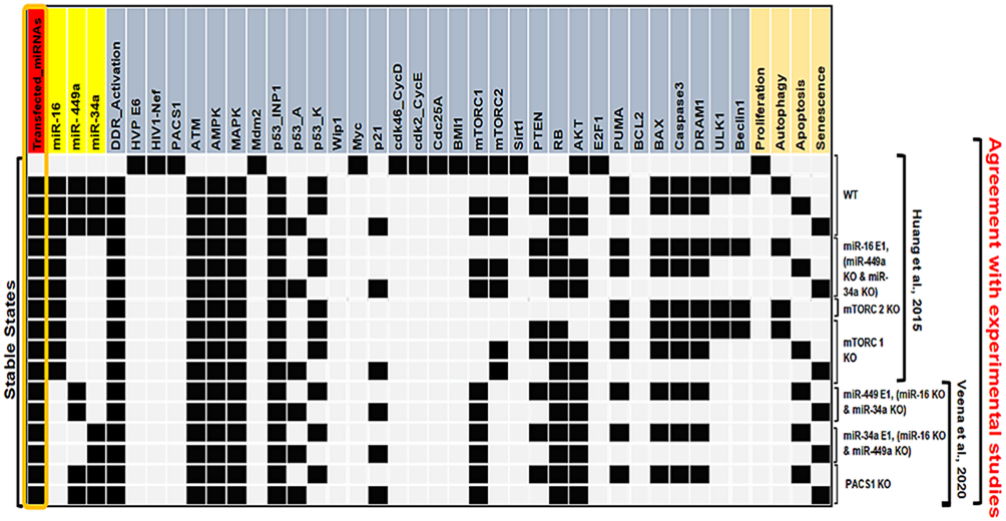
The wild-type state of the synthetic network and experimental verified perturbations. WT, miR-16 E1 along with miR-449a KO and miR-34a KO, mTORC2 KO, mTORC1 KO, miR-449a E1 together with miR-16 KO and miR-34a KO, miR-34a E1 along with miR-449a KO and miR-16 KO, and at the last, PACS1 KO. E1 represents gain-of-function (GoF). Whereas, KO represents loss-of-function (LoF) perturbations corresponding to referential experiments. The left-most column shows levels of Input (Transfected_miRNAs), highlighted in orange color and the right-most column presents the model outputs: Proliferation, Autophagy, Apoptosis and Senescence. Each line represents a single stable state or fixed point corresponding to the input. White cells denote a null i.e., “inactive” value, whereas black cells denote activation means “active” (value 1), respectively.

The network simulations generate four stable states or fixed points for the wild-type case (WT), each of which is associated with a distinct phenotype, as seen in Fig. 3. The first state is a proliferative state (on behalf of the input: Transfected-miRNA = "Inactive"), implying that no G1/S arrest occurs since only cell cycle promoters such as CDK46/CycD, CDK2/CycE, and Cdc25A and HVP E6 and PACS1 are activated. The remaining three states are the result of cell cycle arrest events (also known as tristable dynamics), i.e., they are triggered by a single input: transfected miRNA = "active." The second and third states are associated with cellular death are apoptosis and autophagy, respectively. The second state reflects the autophagy phenotype induced by ULK1/Beclin1 activation in conjunction with DRAM1, whereas, the third stage denotes apoptotic cell death, as long as Caspase3 and DRAM1 are activated, except for ULK1/Beclin1. The fourth stable state depicts the senescence phenotype caused by p53-A and p21 activation.

To determine if these miRNA transfections affected the tristable dynamics, we have performed perturbations of miR-16. We found that ectopic expression of miR-16 (E1) inhibits proliferation and promotes autophagy, apoptosis, and senescence (see in Fig. 3). These findings imply that miR-16 has an effect on the tristable dynamics, which is consistent with Huang et alexperimental's observation of the three phenotypes in HeLa cell cultures^7^.

### miR-449a acts synergistically with miR-34a/miR-16 to regulate the autophagy signaling pathway

To determine how miR-34a and miR-449 transfection can influence senescence and apoptosis in HeLa cells, as indicated by Veena et al.^2^. See Fig. 3, we examine each miRNA independently, which means we knock down (KO) miR-16 in conjunction with miR-34a to elucidate miR-449a's regulatory role. To elucidate the role of miR-34a in the regulation of senescence and apoptosis, we conducted the same thing as previously described, i.e. we knocked down miR-449a in conjunction with miR-16. Following that, we overexpressed both miRNAs (miR-34 E1 and miR-449a E1) in combination with miR-16 knockdown. Finally, we performed a single perturbation of PACS1 by performing a knockdown (KO) of PACS1. We found out that perturbations of miR-34a overexpression (E1) controls senescence, apoptosis, and autophagy in HeLa cells, whereas, overexpression (E1) of miR-449a regulates senescence and apoptosis. Following that, we co-expressed miR-34a and miR-449a E1 to examine their synergistic effects. We observed that miR-449a, in combination with miR-34a, may regulate senescence, autophagy, and apoptosis in HeLa cells following DNA damage at the G1/S checkpoint. Similarly, we overexpressed (E1) miR-16 and miR-449a simultaneously. MiR-449a, in combination with miR-16, was shown to control senescence, autophagy, and apoptosis, as well as a functioning and persistent G1/S checkpoint in HeLa cells. Additionally, we did a PACS1 knockdown (KO) perturbation and discovered that PACS1 KO controls senescence, apoptosis, and autophagy in HeLa cells. (Supplementary File S1).

Furthermore, Veena and colleagues' work^2^ was unable to demonstrate the synergistic regulation of miR-34a and miR-449a in HeLa cells. In this regard, we discovered that each miRNA may control senescence and apoptosis separately (which is entirely by the study of Veena et al.^2^). Additionally, our findings indicate that miR-449a may act in concert with miR-34a or miR-16 to modulate the autophagy signaling pathway. However, miR-449a and miR-34a share a common target^19^, except for mTOR-2^8^, and inhibiting mTOR1/2 expression can induce autophagy in HeLa cells via the ULK1/Beclin1 pathway. Huang et al.^7^ shown that miR-16 targeting mTORC-2 triggers autophagy in Hela cells in this manner. Thus, our findings are consistent with those of Veena et al.^2^ and Huang et al.^7^.

The two experimental investigations mentioned before performed as significant references for developing the model. The technique for developing the model was to align it precisely with the knowledge supplied by each study; for example, one study focused exclusively on one miR-16^7^, ignoring miR-449a/miR-34a. While the other research elucidates the functions of miR-34a and miR-449a but not of miR-16^2^. Additionally, the exact functions of these molecules in the HeLa cell model were explored. To do this, we examined the specific regulatory role of miR-16 in the context of miR-34a and/or miR-449a loss of function (LoF) and similarly examined miR-16 in the context of miR-34a and/or miR-449a loss of function (LoF). For further information, see Fig. 3 and Table 1.

**Table 1:** Agreement between proposed Synthetic Boolean model and experimental data from the literature in HeLa cells.

Stimulus/perturbations	Response/phenotype	References
Transfection of miR-16	Induction of autophagy, apoptosis and senescence	^7^
Knockdown (KO) of mTORC2	Autophagy	^7^
Knockdown (KO) of mTORC1	Induction of autophagy, apoptosis and senescence	^7^
Transfection of miR-449a	Induction of apoptosis and senescence	^2^
Transfection of miR-34a	Induction of apoptosis and senescence	^2^
Knockdown (KO) of PACS1	Induction of apoptosis and senescence	^2^
Synergistic regulation between miR-34 E1 and miR-449a E1	Induction of senescence, autophagy and apoptosis	?
Synergistic regulation between miR-16 E1 and miR-449a E1	Induction of autophagy, apoptosis and senescence	?
Synergistically overexpression (E1) of miR-449a/miR-34a/miR-16	Inhibits proliferation on the functional and stable G1/S checkpoint	?
Targeting of Cdc25A by miR-16/miR-34a/miR-449a	Repression of proliferation through the induction of DNA Damage Response pathways	?
Knockdown (KO) of PACS1	Repression of proliferation through the induction of autophagy along with apoptosis and senescence	?
Transfection of miR-34a	Inhibition of proliferation and induction of senescence, autophagy and apoptosis	?

Ectopic expression (E1) represents gain of function (GoF) (or transfection of miRNA) and Knockdown (KO) represents loss of function (LoF) of the corresponding molecule. Cases for which no experimental data were found are indicated by ‘?’.

When the model is used in conjunction with experimental research, it delivers similar findings as we expected in our work. Then, we proced further to broaden its perturbations, which are currently unknown in the HeLa cell literature as indicated by the question mark (?) in Table 1. For instance, our model predicts that synergistic coordination between miR-34a E1/miR-449a E1 or miR-16 E1/miR-449a E1 can govern HeLa cell senescence, autophagy, and apoptosis. In the second case, transfection of all miRNAs (miR-16 E1/miR-449a E1/miR-34a E1) combined can cause a functioning and persistent G1/S checkpoint. Thirdly, silencing Cdc25A in the presence of miR-16 and/or miR-449a and/or miR-34a can reduce proliferation and induce DNA damage response (DDR) pathways, which can activate a functional and persistent G1/S checkpoint as well as autophagy, apoptosis, and senescence. In the fourth case, silencing/knockdown PACS1 results in autophagy, apoptosis, and senescence. Finally, miR-34a can be used to influence senescence, autophagy, and apoptosis in HeLa cells. Table 1 and Supplementary File S1 include further information.

### Cross-validation through the experimental studies

We estimated in silico the possibilities of each phenotype for the wild-type case when the input (transfection of miRNAs) of the model is ON. We applied the Monte Carlo algorithm in GINsim with 10.000 runs (see section "Methods"). For the WT case, we obtained 60% for senescence, 25% for apoptosis, and 15% for autophagy.

**Figure 4:**
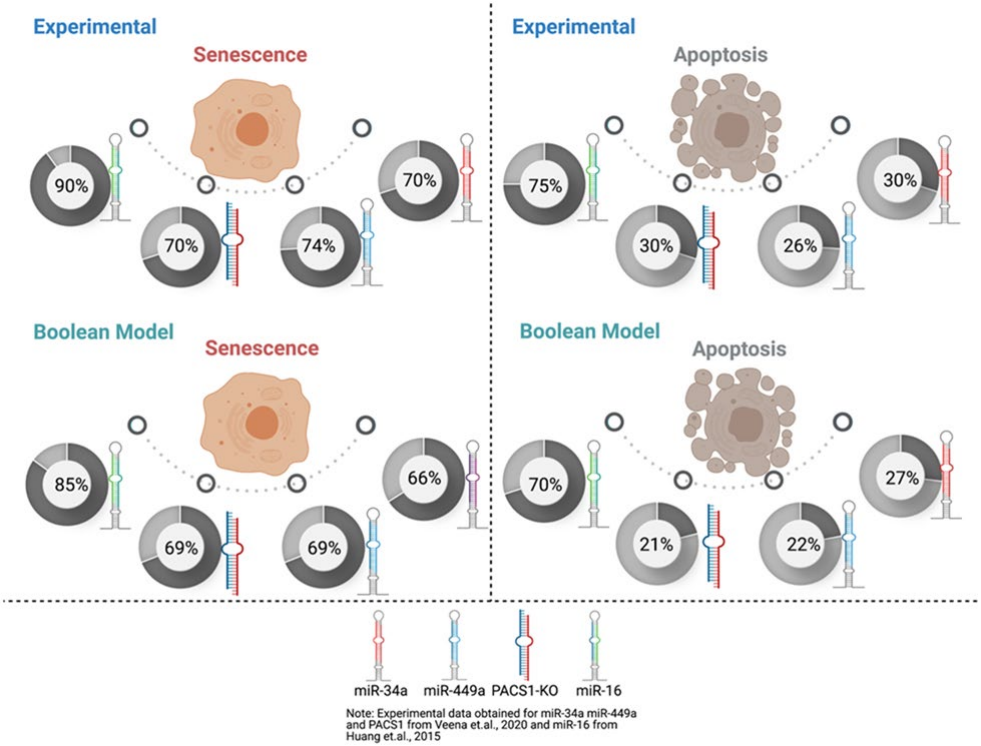
Cross-validation through the experimental studies. Upperside: cell fate decisions such as senescence and apoptosis indicate its corresponding experimental study^2,7^. Whereas, down-side: cell fate decisions such as senescence and apoptosis signify the single node model perturbation. Each circle represents the phenotype percentage that was observed in each experimental study^2,7^. Whereas, the model perturbation for each molecule was obtained through the Monte Carlo simulations (10,000 runs). miR-34a, miR-449a, and miR-16 represent Gain-of-Function (GoF). Whereas, knockdown (KO) represents Loss-of-Function (LoF). For more detail see "Cross-validation through the experimental studies" section.

Additionally, to analyze single node perturbation probabilities, as Veena et al.^2^ and Huang et al.^7^ did in their respective experimental studies we have overexpressed miR-34a and/or miR-449a. They evaluated the performance of these miRNAs by transfection, demonstrating that they accelerated senescence and apoptosis in HeLa cells via PACS1 knockdown (KO). As such, they examine the involvement of miR-449a and/or miR-34a in HeLa cell senescence or apoptosis. Thus, we assessed the probabilities of cell fates such as senescence and apoptosis (Fig. 4) and compared them to those observed in cultures treated for 48 h with miR-34a and/or miR-449a transfection from a subset of HeLa cells^2^. Huang et al.^7^ then employed miR-16 overexpression and knockdown (KO) and investigated the role of this miRNA in the induction of cell fates in HeLa cells by camptothecin (CPT). The MTT test and flow cytometry analysis were used to determine the percentages of cells with senescent and apoptotic phenotypes, respectively. Thus, using our Boolean model, we developed predictions for cell fates such as apoptosis and senescence (Fig. 3) and normalized them to the proportion of Hela cells seen after 36 h of treatment with 10 uM CPT. Additionally, transfection of miR-16 induces G1/S checkpoint arrest when cells are treated for 4 h with hydroxyurea (HU) and propidium iodide (PI). Notably, our model coincides with the findings of Veena et al.^2^ and Huang et al.^7^, Fig. 4.

### Integration of miR-34a/miR-449a/miR-16 on the phenotypic stabilization at the G1/S checkpoint

**Figure 5:**
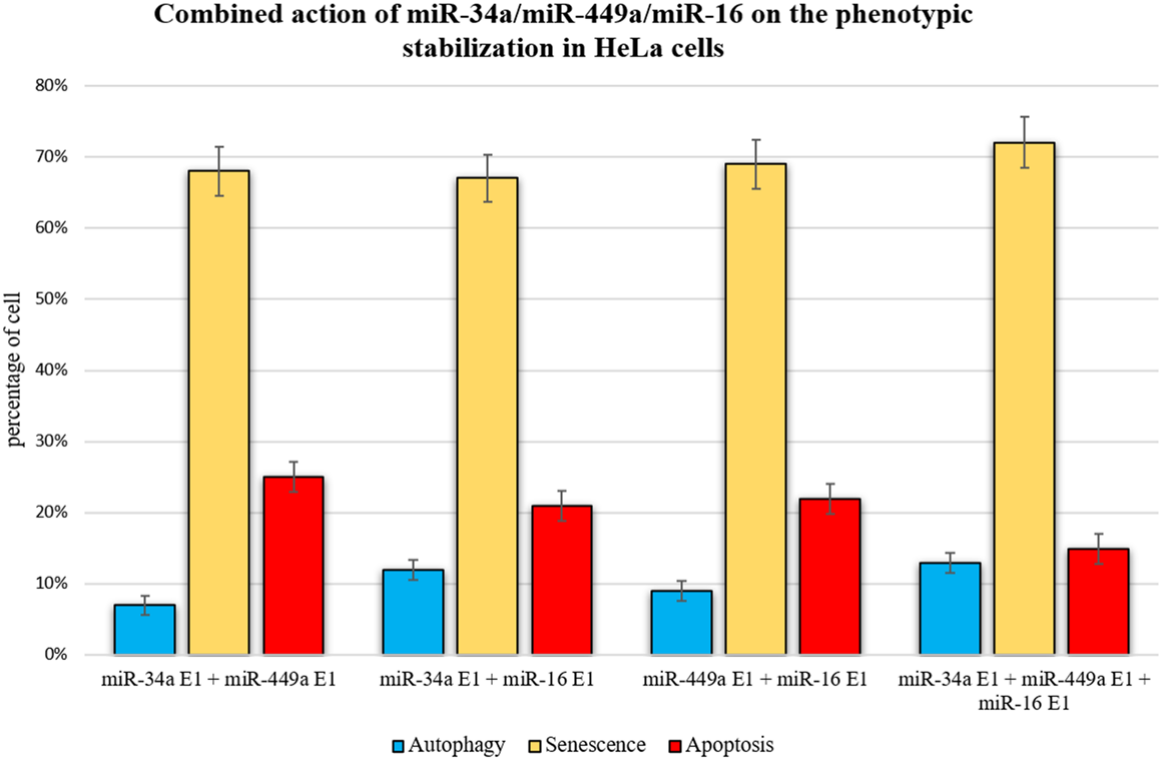
Integration of miR-34a, miR-449a, and miR-16 on the phenotypic stabilization in HeLa cells. The First synergy case between overexpression (E1) of miR-34a E1/miR-449a E1. The second synergy case between miR-34 E1/miR-16 E1 overexpression (E1). Whereas, the third case between miR-449a E1/miR-16 E1. At the last, all these miRNAs overexpressed (E1) together i.e., miR-34a E1/miR-449a E1/miR-16 E1. Each particular color bar denotes its corresponding phenotype. For each case, we have run 10.000 Monte Carlo simulations. In addition, the first three cases are compared with the last case of synergy. For more detail see "Integration of miR-34a/miR-449a/miR-16 on the phenotypic stabilization at the G1/S checkpoint" section.

Subsequently, we examined whether the combination of these miRNAs had an effect on phenotype stabilization at the G1/S checkpoint. To do so, we performed a single perturbation, in which we over-expressed (E1) all of these miRNAs simultaneously (miR-34a E1 + miR-449a E1 + miR-16 E1) and compared it to combined cases of miRNAs, such as (miR-34a E1 + miR-449 E1), (miR-34a E1 + miR-16 E1) and (miR-449a E1 + miR-16 E1). We perturbed the gain of function (GoF) in each example and ran Monte Carlo simulations with 10,000 runs (see section "Methods"). This allowed us to investigate all conceivable interactions between these miRNAs (Fig. 5). We observed that miR-34a E1/miR-449a E1 combined action generated the most prominent apoptotic phenotype and decreased the autophagic phenotype. MiR-34a E1/miR-16 E1 acting in concert enhanced the autophagic phenotype. MiR-16 E1/miR-449a E1 regulation improves the senescent phenotype in cells but has a little effect on autophagy. Finally, a combination of all three miRNAs induces an increase in the number of senescent cells, increases the autophagic phenotype, and decreases the apoptotic phenotype.

### ATM: the main controller of multiple DNA damage response signaling pathways

**Figure 6:**
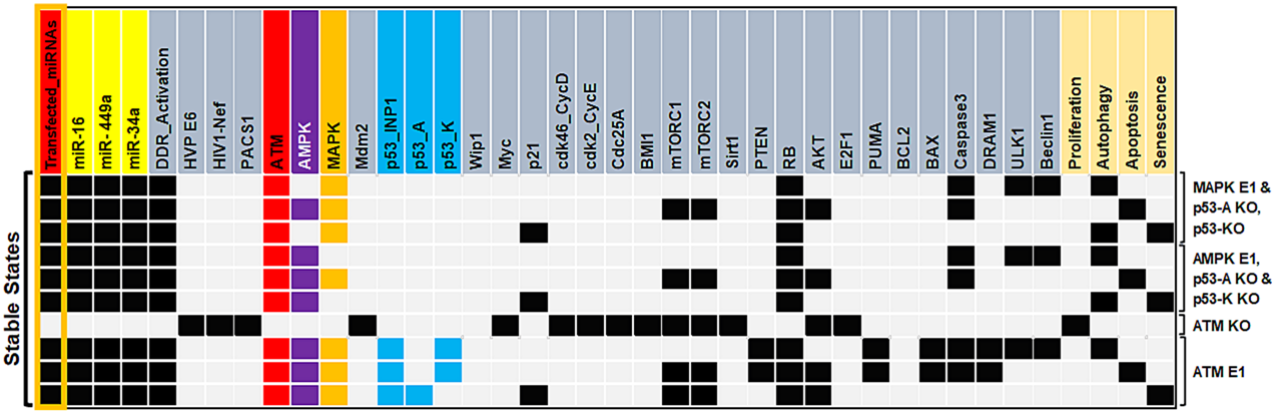
The p53-independent signaling pathways. Gain of function (GoF) determines overexpression (E1) while loss of function (LoF) describes the knockout (KO) of the component corresponding to the model. The stable states were characterized for several scenarios: MAPK E1, AMPK E1 along with p53 KO, ATM KO, and ATM E1. White cells indicate a zero value, while black/red/purple/orange and blue cells indicate activation (value 1). The left-hand side highlighted in orange box presents the status of the input transfected miRNAs and the right-hand side shows the outputs of the model such as proliferation, autophagy, apoptosis, and senescence. Per line describes a single stable state or fixed point analogous to the input. The first three steady states or fixed points belong to the gain-of-function (GoF) of MAPK as well as loss-of-function (LoF) of p53 and the next three steady states describe the gain-of-function (GoF) of AMPK along with the loss-of-function (LoF) of p53: defines alternative signaling pathways in the G1/S arrest. The knockout (KO) of ATM inhibits arrest. Whereas, the gain-of-function (GoF) of ATM suppresses proliferation and induces the identified arresting phenotypes such as autophagy, apoptosis, and senescence.

It is widely established that the p53 pathway is a critical regulator of cell fate decisions in DDR^20^. Numerous studies published recently have suggested that the MAPK or AMPK signaling pathways play a substantial role (independent of p53) in inducing cell fate in cervical cancer^21,22^. As a result, we inquire if miR-16, miR-34a, and miR-449a transfection may control additional signaling pathways such as MAPK or AMPK (in the absence of p53) (see Fig. 6). We observed that overexpression (E1) of either MAPK or AMPK may control autophagy, apoptosis, and senescence at the functional G1/S checkpoint. Our findings concur with those of Zhong et al.^23^ and Law et al.^24^, indicating that alternative pathways such as MAPK and/or AMPK may be responsible for cell destiny in HeLa cells in a p53-independent manner. Additionally, the analysis revealed that these perturbations are consistent with the Taji et al. study's^25^ observation of an interchangeable double-phenotype state (senescence + autophagy) in HeLa cells^25^. Thus, our data indicate that transfection of miR-16, miR-34a, and miR-449a into Hela cells may activate the G1/S checkpoint (with or without p53) via numerous signaling pathways such as AMPK or MAPK.

Moreover, current research indicates that ATM can control senescence, autophagy, and apoptosis, as well as various DNA damage signaling pathways. For example, Liang et al.^26^ had shown that the ATM is necessary to trigger autophagy and apoptosis in HeLa cells via the MAPK pathway. Furthermore, they found that silencing ATM abolishes autophagy and apoptosis while promoting cancer development whereas, overexpression of ATM suppresses proliferation. Beauvarlet et al.^27^, revealed that ATM is essential to regulate the balance of senescence, autophagy, and apoptosis in HeLa cells via the ATM/AMPK pathway (independent of p53). Sanli et al.^28^ revealed that the ATM/AMPK pathway may control autophagy and apoptosis in HeLa cells in the absence of p53. On the other side, ATM deficiency increases the proliferation^28^. We tested it, as seen in Fig. 6, by knocking down ATM and observed that knocking down ATM increases the proliferation, while overexpression of ATM inhibits proliferation and regulates autophagy, apoptosis, and senescence in HeLa cells at the G1/S phase. Our findings are highly consistent with those of these investigations^26–28^. To summarize, ATM is the primary regulator of DDR's various signaling pathways (Fig. 6).

## Discussion

In this study, we have investigated the molecular mechanisms by considering the performance of miR-16, miR-34a, and miR-449a individually or synergistically at the functional G1/S checkpoint in HeLa cells (see Fig. 2). Recently, Veena et al.^2^ found that PACS1, which is the direct target of miR-34a and miR-449a is upregulated in HeLa cells and promotes cancer progression while observing the decreased expression of miR-34a and miR-449a in the HeLa cells, simultaneously. They have also reported that the transfection of miR-34a and/or miR-449a inhibits cancer proliferation by the induction of senescence and apoptosis in HeLa cells. The aforementioned study shows that manipulation of miR-34a and miR-449a can modulate senescence and apoptosis in a p53-dependant manner in HeLa cells. Previously, Huang et al.^7^ demonstrated that the transfection of miR-16 suppressed cell proliferation and promoted autophagy, apoptosis, and senescence by targeting mTOR at the G1/S checkpoint in HeLa cells.

The synergistic coordination among the miR-16, miR-34a, and miR-449a in the functional G1/S checkpoint and the molecular mechanisms involving these miRNAs in cell fate decisions for HeLa cells is challenging. For that, a Boolean model was constructed based on the available experimental data^2,7^ in the HeLa cells. In the lack of transfection of miRNAs, the model predicts the only proliferative state, which is consistent with experimental studies^2,7^. In the presence of miRNAs (transfection of miRNAs), the model predicts autophagy, apoptosis, and senescence, which is agreeable with Huang et al.^7^ (See Fig. 3).

Likewise, we demonstrated the regulatory role of individual miRNAs or synergistically (with two or more miRNAs) in HeLa cells' functioning G1/S checkpoint. To do this, we examined each miRNA's perturbations, including ectopic (E1) and knockdown (KO) expression (see inTable 1). Individual transfections of miR-16 or miR-34a can elicit tristable dynamics (autophagy, apoptosis, and senescence) as well as a functioning and stable G1/S checkpoint, whereas miR-449a regulates only bistability (senescence and apoptosis) but not autophagy. Transfection of miR-449a in combination with miR-16 and/or miR-34a can induce tristable dynamics in a synergistic way (for more details see Table 1 and Supplementary File S1). Indeed, Veena and colleagues' investigation did not demonstrate a synergistic regulation between miR-34a and miR-449a in HeLa cells^2^. We found out that each miRNA (miR-34a and miR-449a) may control senescence and apoptosis separately. Additionally, our data indicated that miR-34a controls the autophagy signaling pathway by targeting mTOR alone (or in conjunction with miR-449a/miR-16). Similarly, miR-449a can regulate the autophagy signaling pathway in conjunction with miR-34a or miR-16. Indeed, miR-449a and miR-34a share common targets^19^, but miR-34a has a unique target in mTOR-2^8^. It has been demonstrated that silencing mTOR1/2 expression enhances the induction of autophagy by ULK1/Beclin1 in HeLa cells^29^. Similarly, Huang et al.^7^ demonstrated that miR-16-mediated silencing of mTORC-2 controls autophagy in Hela cells. Thus, our findings are consistent with those of Veena et al.^2^ and Huang et al.^7^.

Additionally, after the model cooperates with experimental research, we opted to increase its perturbations in HeLa cells that are still unknown. For example, our model predicts that synergistic co-expression of miR-34a E1/miR-449a E1 or miR-16 E1/miR-449a E1 can influence autophagy, apoptosis, and senescence in HeLa cells. In the second case, co-transfection of all miRNAs (miR-16 E1/miR-449a/miR-34a E1) can establish a functioning and persistent G1/S checkpoint. The third prediction is that silencing Cdc25A in the presence of miR-16 and/or miR-449a and/or miR-34a can inhibit proliferation and thus the induction of DNA Damage Response pathways, which may result in the activation of a functional and stable G1/S checkpoint, as well as autophagy, apoptosis, and senescence. In the fourth case, silencing PACS1 results in autophagy, apoptosis, and senescence.

Veena et al. showed that knocking down PACS1 promoted apoptosis and senescence in HeLa cells. In light of this, we hypothesize that knocking down PACS1 can influence autophagy as well as senescence and apoptosis. Furthermore, we performed cross-validation on each experimental trial. We ran a Monte Carlo simulation (10.000 times) and compared the results to those of Veena et al.^2^ and Huang et al.^7^. These findings are entirely consistent with the findings of these investigations^2,7^. (see Fig. 4). Additionally, we studied three unique instances of synergistic overexpression (E1) between these miRNAs (miR-34a E1/miR-449a E1), (miR-449a E1/miR-16 E1), and (miR-34a E1/miR-16 E1), and compared them to the case of all three miRNAs combined (miR-34a E1/miR-449a E1/miR-16 E1). This enables us to deduce the specific effect of those miRNAs acting in synergy on phenotypic stability. The results are shown in Fig. 5. Each instance of the synergistic relationship illustrates the distinct consequences of phenotypes. For instance, miR-34a E1/miR-449a E1 increased apoptosis and decreased autophagy. MiR-34a E1/miR-16 E1 co-expression increased the autophagic phenotype. Mutual regulation of miR-449a E1/ miR-16 E1 increases senescent phenotypes in cells but has a little effect on autophagy. Finally, integration of all three miRNAs (miR-34a E1 + miR-449a E1 + miR-16 E1) results in an increase in senescent cells, an increase in the autophagic phenotype, and a decrease in the apoptotic phenotype (Fig. 5).

**Figure 7:**
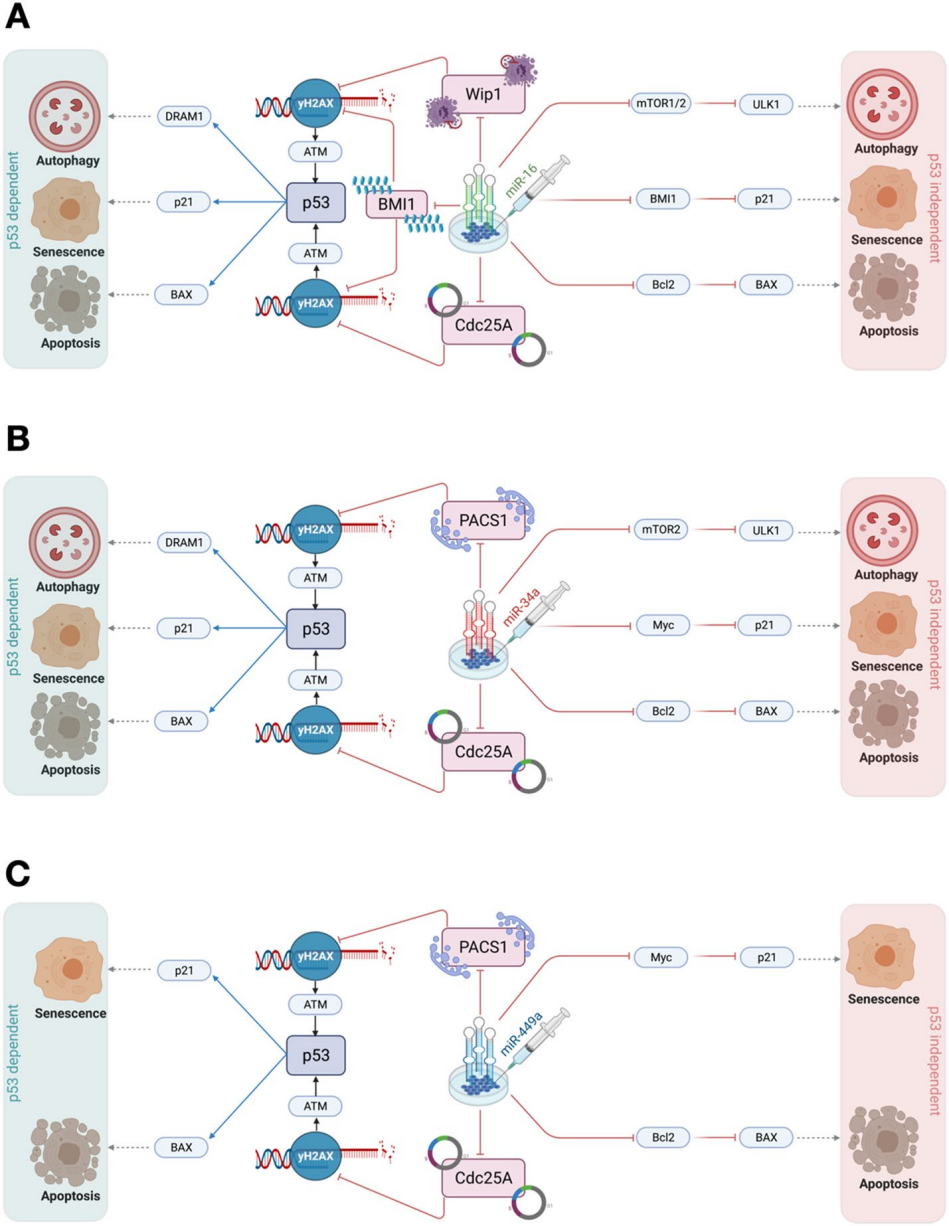
Comprehensive molecular mechanisms of transfection of miRNAs on tumor growth and proliferation in HeLa cells. (**A**) Transfection of miR-16-induced DNA damage in cells by targeting Wip1^6^ and Cdc25A^3^. Once DNA damage is triggered in cells it may regulate p53-dependent or independent molecular mechanisms at the functional G1/S checkpoint^20^. miR-16 directly targets mTOR1/2 induced autophagic cell death^7,60^. Whereas miR-16 controls the senescent phenotype by targeting BMI1, which regulates p21 expression^61^. Whereas, miR-16 regulates apoptotic cell death by targeting Bcl2, which triggers BAX/Caspase expression^62^. On the other hand, transfection of miR-16 regulates cell fate through the p53^46^. miR-16 accelerated the p53 pathway and then, p53 induces autophagic cell death through DRAM1^63^. Senescence by induction of p21^20^ while apoptotic through induction of BAX/Caspase^20^. (**B**) Transfection of miR-34-induced DNA damage in cells by targeting PACS1 and Cdc25A^2,64^. Once DNA damage is triggered in cells it may control p53-dependent or independent molecular mechanisms at the functional G1/S checkpoint. miR-34 directly targets mTOR2 induced autophagic cell death^8^. Whereas miR-34a regulates the senescent phenotype by targeting Myc^65^, which regulates p21 expression. Whereas, miR-34a rules apoptotic cell death by targeting Bcl2^66^, which triggers BAX/Caspase expression. On the other hand, transfection of miR-34 coordinates cell fate through the p53^34^. miR-34 stimulated the p53 pathway and then, p53 induces autophagic cell death through DRAM1^63^. Senescence by induction of p21^20^ while apoptotic through the introduction of BAX/Caspase^20^. (**C**) Transfection of miR-449a-induced DNA damage in cells by targeting PACS1 and Cdc25A^2,45^. Once DNA damage is generated in cells it may control p53-dependent or independent molecular mechanisms at the functional G1/S checkpoint^2^. miR-449a controls the senescent phenotype by targeting Myc^67^, which modulates p21 expression. Whereas, miR-449a rules apoptotic cell death by targeting Bcl2^68^, which triggers BAX/Caspase expression. On the other hand, transfection of miR-449a regulates cell fate through the p53^2^. miR-449a activated the p53 pathway and then, p53 induces senescence by the activation of p21^20^, while triggers the apoptosis by the activation of BAX/Caspase^20^. On the right, p53-independent molecular mechanisms of cell fate decisions such as autophagy, senescence, and apoptosis. On the left, p53-dependent molecular mechanisms of cell fate determination (autophagy, senescence, and apoptosis). Red-hammer head arrows represent inhibitions, while blue arrows indicate activation, respectively.

**Figure 8:**
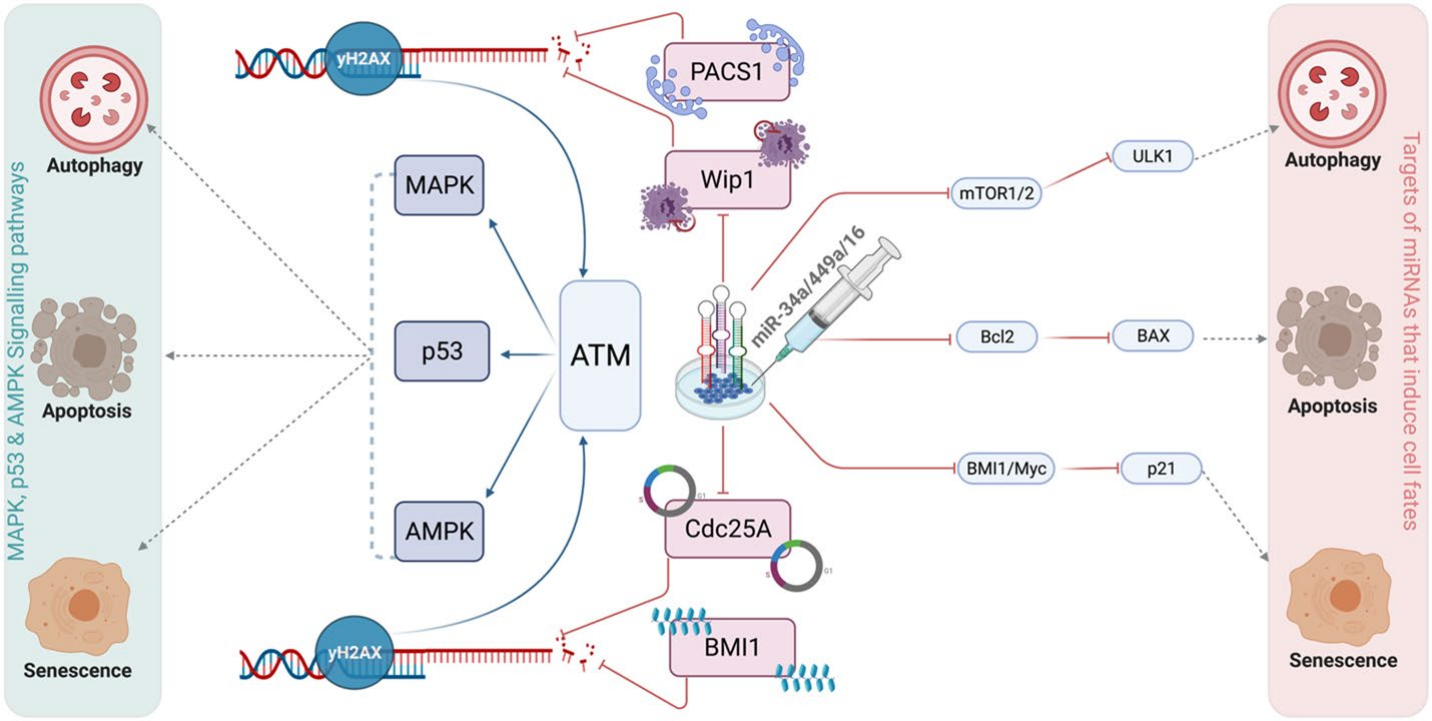
Schematic illustration depiciting the role of three miRNAs (miR-34a, miR-449a, and miR-16) at the G1/S checkpoint which can modulate multiple DDR signaling pathways in HeLa cells.

Likewise, in the absence of p53, transfection of miR-16, miR-34a, and miR-449a can influence phenotypes such as autophagy, apoptosis, and senescence. Notably, we inferred that the MAPK and/or AMPK signaling pathways can modulate the functional G1/S checkpoint (when the p53 pathway is not functional in the cells). Additionally, we observed that ATM is required for the functioning G1/S checkpoint in HeLa cells, as well as for the regulation of autophagy, apoptosis, and senescence, as proposed by Liang et al.^26^ (see Fig. 6). Thus, our findings suggest that manipulating these miRNAs can activate the functional G1/S checkpoint, as well as autophagy, apoptosis, and senescence, via MAPK or AMPK pathways (independent of p53). For more detail see Fig. 7. Thus, this is the first and most comprehensive work to explore the involvement of three miRNAs (miR-34a, miR-449a, and miR-16) at the G1/S checkpoint and to demonstrate that these miRNAs may influence numerous DDR signaling pathways in cervical cancer (see in Fig. 8).

In conclusion, our model agrees with experimental results concerning the individual effect of miRNA(s) in cell fate decisions in HeLa cells. We shed light on a very complex landscape between these three miRNAs. Furthermore, our testable predictions abolished HeLa cancer progression through the induction of functional and stable G1/S checkpoint. Therefore, our synthetic approach may contribute to the inhibition of tumor growth and proliferation in cancer cells through the transfection of these miRNAs.

## Methods

### Collection of the public databases/tool and structure of the Gene Regulatory Network in HeLa cells

Developing a gene regulatory network concerning miRNAs (miR-34a, miR-449a, and miR-16). We have only used PubMed studies and databases such as BIOGRID 3.5 (https://thebiogrid.org/)^30^. The focus was to identify genes or proteins that were targeted by these miRNAs (Fig. 2) such as Cyclin-dependent kinases 4 and 6 complexes/CyclinD1 (CDK4/6-Cyclin D), Cyclin-dependent kinase 2/CyclinE2 (CDK2/Cyclin E), Cell division cycle 25A (Cdc25A), E2F1, and BCL2, are the experimentally verified common targets of miR-34a^31^, miR-449a^32^ and miR-16^33^. In addition, Myc, Sirt1 and PACS1 are unique targets of miR-34a^2,34^ and miR-449a^2,32^, except miR-16. Whereas, different targets of miR-16 are BMI1, Wip1, and mTOR-1/2^33^. Whereas, the unique target of miR-34a is mTOR 2^8^. For that, we have used public databases like; TARGET SCAN HUMAN 7.2 (http://www.targetscan.org/vert_72/)^35^.

GINsim 3.0.0b was used for the construction and simulation of the Boolean model and visualization of the results^36^. Which is a Java-based software and is freely available to researchers (http://www.ginsim.org/downloads)^36^. GINsim algorithms recognize all the attractors for the wild-type case (unpertubated Boolean model) as well as for various mutant situations. The model file is available in the "Code Availability" section.

### PubMed literature based boolean network, rules and simulations

The Boolean method is based on the characterization of a regulatory graph, where an individual node defines a molecule and each directed edge (or arc) signifies an activation or inhibition among two nodes. Nodes are Boolean variables that only consider “0” and “1” i.e., “active” and “inactive” values. Based on the description of the biochemical information, each node in the network is assigned a logical rule, which determines its activation level concerning the position of its regulators^37^.

A Boolean network of miRNAs (miR-34a, miR-449a, and miR-16) was generated by translating the biological interactions described in the gene regulatory network (Fig. 2) into Boolean rules. These Boolean rules regulating nodes are based on the biochemical literature from PubMed and are available in Supplementary Table S1. Classical Boolean operators were used to write these rules "AND, "OR" and "NOT". Attractors are the main outcome of simulations using a Boolean network. The dynamical performance of a Boolean model can be interpreted by a state transition graph (STG). In this graph, each node describes the state of the network variables and the arcs signify transitions between these states. The STG serves all possible trajectories that one initial state can drive to a final state. Terminal nodes that have no outgoing edges are called stable states (or fixed points) while a set of transitions trapped among a fixed group of states in the STG defines a cyclic state. For the updates of states, asynchronous updates were considered^38^, which has the potential to describe non-deterministic behavior observed in molecular networks. Moreover, this approach allows in silico gain-of-function (GoF) or loss-of-function (LoF) perturbations, we force node values to be “active” or “inactive”, respectively, to examine the effect of particular nodes on network dynamics and the resulting phenotype^36,37^.

### miRNAs-mediated molecular mechanisms at the G1/S checkpoint in HeLa

Cell-fate determination such as apoptosis, senescence or autophagy occur at cell cycle checkpoints^20^. As the G1/S checkpoint is p53 pathway-dependent and it is caused via DNA Damage, we added a new layer of complexity to the operative G1/S regulatory network based on the works by Xie et al.^39^ and Huang et al.^7^ who considered DNA-damage induced autophagy in Hela cells. Here we briefly outline the central direct molecular interactions known in the literature that constitute our synthetic regulatory network.

The studies of Veena et al.^2^ and Huang et al.^7^ used miR-34a and miR-449a and miR-16 transfection to induce autophagy, apoptosis, and senescence. Transfection of miRNAs leads to a G1/S checkpoint activation for suppressing Wip1, Cdc25A, BMI1 and PACS1^2,3,6,40^. Then in our synthetic network, we used all these as an input signal (see Fig. 2). In addition, miR-34a, miR-449a, and miR-16 transfection induce a DNA-damage response in Hela cells activating the ATM/p53 pathway^2,7^. which are involved in the induction of senescence, apoptosis, and autophagy^2,7^. The targets of miR-34a, miR-449a and miR-16 in the network are presented in what follows.

DNA double-strand breaks generated by radiomimetic chemicals or reactive oxygen species (ROS), drugs or ionizing radiation can stimulate the ATM and p53 pathways^41^. ATM or p53 can directly induce miR-34a^42,43^ and stimulated p53 triggers the transcription of the E3 ubiquitin-protein ligase (Mdm2) which is its own negative regulator^44^. Whereas, miR-449a can be induced by DNA Damage^45^. As in our previous models (for more details see^46–51^), based on the several phosphorylation events that p53 possesses, it is described by two variables: p53-A and p53-K. P53-A serves p53 phosphorylated at serine 15 and serine 20, whereas p53-K describes additional phosphorylation at serine 46, in which case p53 becomes an inducer of apoptosis. P21 (Cyclin-dependent kinase inhibitor 1A), Wip1, and tumor protein p53 inducible nuclear protein 1 (TP53INP1) are initiated by p53-A and p53-K. ATM serine/threonine kinase (ATM) phosphorylates p53 to the p53-A form that initiates transcription of Wip1, which in turn inactivates ATM^6^. Similarly, the apoptosis controller Bcl-2-binding component 3 (BBC3, also called PUMA), DRAM1, and the BCL2 associated X (BAX) are initiated by p53-K. In the model, the apoptotic phenotype entails activation of Caspase 3 through the BAX which is ruled by PUMA and DRAM1.Whereas, p21 activation is correlated with the senescence phenotype^52^. It recognized that the targeting of mTOR Complex 1 (mTORC1) and mTOR Complex 2 (mTORC2) can begin the autophagy phenotype^53^. mTOR directly represses the Unc-51-like kinase 1 (ULK1) protein complex, which plays a fundamental role in provoking autophagy^53,54^. Interference of the mTORs expression intensifies ULK1/Beclin-1 activity leading to autophagy induction^55^.

DRAM1 is needed to induced apoptosis via direct activation of BAX^56^. On the other hand, DRAM1 represses mTORs expression, which indirectly activates ULK1/ Beclin-1^57^. Thus, p53 coordinates apoptotic/autophagic phenotypes^39^. CDK4/6-Cyclin D and CDK2/Cyclin E are the foremost cell cycle regulators that boost the G1 to S phase transition^58^.

Based on the main interactions above we defined our Boolean model of the G1/S checkpoint regulation in Hela cells (Fig. 2).

### Expected performances of tumor suppressor miRNAs in HeLa cell line

Simulations using the miRNAs (miR-34a, miR-449a, and miR-16) in the Boolean model should describe the cancer biology of the cell. Indeed, cancer cells can be uncomplicated as they either remain in a proliferative state due to downregulation of miR-34a, miR-449a and miR-16 i.e., upregulation of PACS1 or AKT/mTOR pathway (in the absence of DNA damage or treatment). Whereas in the case of DNA damage i.e., when DNA damage is present in cancer cells, upregulation of these tumors suppressors miR-34a, miR-449a, and miR-16 can inhibit the tumor growth and proliferation through the induction of senescence and/or cell deaths such as apoptotic or autophagic at the G1/S checkpoint. Therefore, at least four fixed points/stable states (attractors) are expected.

### Statistical information

GINsim 3.0.0b is a powerful tool^36^ and recently introduced three different algorithms to estimate their reachability probabilities: Monte Carlo, Avatar, and Firefront. In this work, we have used "Monte Carlo simulation" because of its ability to define the reachability probabilities of the Boolean model attractors under an asynchronous updating scheme^59^. Monte Carlo is just as fast and effective as Avatar or Firefront for estimating the probabilities of reaching steady states. Avatar and Firefront are more valuable for dealing with cyclic attractors. However, since no cyclic attractors were identified in our Boolean network simulations. So, we chose Monte Carlo over Avatar and Firefront. Therefore, GINsim allows the estimation of probabilities to reach specific attractors. In this work, we adopted Monte Carlo simulations with 'exact exit probabilities' and compared them with clinical outcomes^2,7^.

## Supplementary Information

Supplementary Information 1.

Supplementary Information 2.

## Data Availability

All data needed to evaluate the conclusions in the paper are present in the paper and/or the Supplementary Materials.

## Code availability

The model code is available in the Github repository: https://github.com/pritampanda15/Dynamic-modeling-of-miR-34a-miR-449a-and-miR-16......HeLa-cells.

## References

[1] Wang Y, Taniguchi T (2013). MicroRNAs and DNA damage response. Cell Cycle.

[2] Veena MS, Raychaudhuri S, Basak SK, Venkatesan N, Kumar P, Biswas R, Chakrabarti R, Lu J, Su T, Gallagher-Jones M, Morselli M, Fu H, Pellegrini M, Goldstein T, Aladjem MI, Rettig MB, Wilczynski SP, Shin DS, Srivatsan ES (2020). Dysregulation of hsa-miR-34a and hsa-miR-449a leads to overexpression of PACS-1 and loss of DNA damage response (DDR) in cervical cancer. J. Biol. Chem..

[3] Pothof J, Verkaik NS, Van Ijcken W, Wiemer EA, Ta VT, Van Der Horst GT, Jaspers NG, Van Gent DC, Hoeijmakers JH, Persengiev SP (2009). MicroRNA-mediated gene silencing modulates the UV-induced DNA-damage response. EMBO J..

[4] Wang H-Y, Liu Z-S, Qiu L, Guo J, Li Y-F, Zhang J, Wang T-J, Liu X-D (2014). Knockdown of Wip1 enhances sensitivity to radiation in HeLa cells through activation of p38 MAPK. Oncol. Res..

[5] Choi DW, Na W, Kabir MH, Yi E, Kwon S, Yeom J, Ahn J-W, Choi H-H, Lee Y, Seo KW, Shin MK, Park S-H, Yoo HY, Isono K-I, Koseki H, Kim S-T, Lee C, Kwon YK, Choi CY (2013). WIP1, a homeostatic regulator of the DNA damage response, is targeted by HIPK2 for phosphorylation and degradation. Mol. Cell.

[6] Shreeram S, Demidov ON, Hee WK, Yamaguchi H, Onishi N, Kek C, Timofeev ON, Dudgeon C, Fornace AJ, Anderson CW (2006). Wip1 phosphatase modulates ATM-dependent signaling pathways. Mol. Cell.

[7] Huang N, Wu J, Qiu W, Lyu Q, He J, Xie W, Xu N, Zhang Y (2015). MiR-15a and miR-16 induce autophagy and enhance chemosensitivity of Camptothecin. Cancer Biol. Ther..

[8] Rathod SS, Rani SB, Khan M, Muzumdar D, Shiras A (2014). Tumor suppressive miRNA-34a suppresses cell proliferation and tumor growth of glioma stem cells by targeting Akt and Wnt signaling pathways. FEBS Open Bio.

[9] Torossian A, Broin N, Frentzel J, Daugrois C, Gandarillas S, Saati TA, Lamant L, Brousset P, Giuriato S, Espinos E (2019). Blockade of crizotinib-induced BCL2 elevation in ALK-positive anaplastic large cell lymphoma triggers autophagy associated with cell death. Haematologica.

[10] Shan C, Chen X, Cai H, Hao X, Li J, Zhang Y, Gao J, Zhou Z, Li X, Liu C, Li P, Wang K (2021). The emerging roles of autophagy-related microRNAs in cancer. Int. J. Biol. Sci..

[11] Hall DP, Cost NG, Hegde S, Kellner E, Mikhaylova O, Stratton Y, Ehmer B, Abplanalp WA, Pandey R, Biesiada J, Harteneck C, Plas DR, Meller J, Czyzyk-Krzeska MF (2014). TRPM3 and miR-204 establish a regulatory circuit that controls oncogenic autophagy in clear cell renal cell carcinoma. Cancer Cell.

[12] He C, Klionsky DJ (2009). Regulation mechanisms and signaling pathways of autophagy. Annu. Rev. Genet..

[13] Chowdhari S, Saini N (2016). Gene expression profiling reveals the role of RIG1 like receptor signaling in p53 dependent apoptosis induced by PUVA in keratinocytes. Cell Signal.

[14] Hietanen S, Lain S, Krausz E, Blattner C, Lane DP (2000). Activation of p53 in cervical carcinoma cells by small molecules. PNAS.

[15] Al-Mohanna MA, Al-Khodairy FM, Krezolek Z, Bertilsson P-A, Al-Houssein KA, Aboussekhra A (2001). p53 is dispensable for UV-induced cell cycle arrest at late G1 in mammalian cells. Carcinogenesis.

[16] Gao J, Yu H, Guo W, Kong Y, Gu L, Li Q, Yang S, Zhang Y, Wang Y (2018). The anticancer effects of ferulic acid is associated with induction of cell cycle arrest and autophagy in cervical cancer cells. Cancer Cell Int..

[17] Hsieh WT, Lin HY, Chen JH, Kuo YH, Fan MJ, Wu RSC, Wu KC, Wood WG, Chung JG (2011). Latex of euphorbia antiquorum induces apoptosis in human cervical cancer cells via c-Jun N-terminal kinase activation and reactive oxygen species production. Nutr. Cancer.

[18] Chen Y-H, Yang S-F, Yang C-K, Tsai H-D, Chen T-H, Chou M-C, Hsiao Y-H (2021). Metformin induces apoptosis and inhibits migration by activating the AMPK/p53 axis and suppressing PI3K/AKT signaling in human cervical cancer cells. Mol. Med. Rep..

[19] Lizé M, Klimke A, Dobbelstein M (2011). MicroRNA-449 in cell fate determination. Cell Cycle.

[20] Hafner A, Bulyk ML, Jambhekar A, Lahav G (2019). The multiple mechanisms that regulate p53 activity and cell fate. Nat. Rev. Mol. Cell Biol..

[21] Li J, Jiang P, Robinson M, Lawrence TS, Sun Y (2003). AMPK-beta1 subunit is a p53-independent stress responsive protein that inhibits tumor cell growth upon forced expression. Carcinogenesis.

[22] Reinhardt HC, Aslanian AS, Lees JA, Yaffe MB (2007). p53 deficient cells rely on ATM and ATR-mediated checkpoint signaling through the p38 MAPK/MK2pathway for survival after DNA damage. Cancer Cell.

[23] Zhong W, Zhu H, Sheng F, Tian Y, Zhou J, Chen Y, Li S, Lin J (2014). Activation of the MAPK11/12/13/14 (p38 MAPK) pathway regulates the transcription of autophagy genes in response to oxidative stress induced by a novel copper complex in HeLa cells. Autophagy.

[24] Law BYK, Mok SWF, Chan WK, Xu SW, Wu AG, Yao XJ, Wang JR, Liu L, Wong VKW (2016). Hernandezine, a novel AMPK activator induces autophagic cell death in drug-resistant cancers. Oncotarget.

[25] Taji F, Kouchesfahani HM, Sheikholeslami F, Romani B, Baesi K, Vahabpour R, Edalati M, Teimoori-Toolabi L, Jazaeri EO, Abdoli A (2017). Autophagy induction reduces telomerase activity in HeLa cells. Mech. Ageing Dev..

[26] Liang N, Jia L, Liu Y, Liang B, Kong D, Yan M, Ma S, Liu X (2013). ATM pathway is essential for ionizing radiation-induced autophagy. Cell. Signal..

[27] Beauvarlet J, Bensadoun P, Darbo E, Labrunie G, Rousseau B, Richard E, Draskovic I, Londono-Vallejo A, Dupuy J-W, Nath Das R, Guédin A, Robert G, Orange F, Croce S, Valesco V, Soubeyran P, Ryan KM, Mergny J-L, Djavaheri-Mergny M (2019). Modulation of the ATM/autophagy pathway by a G-quadruplex ligand tips the balance between senescence and apoptosis in cancer cells. Nucleic Acids Res..

[28] Sanli T, Steinberg GR, Singh G, Tsakiridis T (2014). AMP-activated protein kinase (AMPK) beyond metabolism: A novel genomic stress sensor participating in the DNA damage response pathway. Cancer Biol Ther.

[29] Xu T, Sun D, Chen Y, Ouyang L (2020). Targeting mTOR for fighting diseases: A revisited review of mTOR inhibitors. Eur. J. Med. Chem..

[30] Chatr-Aryamontri A, Oughtred R, Boucher L, Rust J, Chang C, Kolas NK, O’Donnell L, Oster S, Theesfeld C, Sellam A (2017). The BioGRID interaction database: 2017 update. Nucleic Acids Res..

[31] Zhang L, Liao Y, Tang L (2019). MicroRNA-34 family: A potential tumor suppressor and therapeutic candidate in cancer. J. Exp. Clin. Cancer Res..

[32] Yong-Ming H, Ai-Jun J, Xiao-Yue X, Jian-Wei L, Chen Y, Ye C (2017). miR-449a: A potential therapeutic agent for cancer. Anticancer Drugs.

[33] Aqeilan RI, Calin GA, Croce CM (2010). miR-15a and miR-16-1 in cancer: Discovery, function and future perspectives. Cell Death Differ..

[34] Slabáková E, Culig Z, Remšík J, Souček K (2017). Alternative mechanisms of miR-34a regulation in cancer. Cell Death Disease.

[35] Agarwal V, Bell GW, Nam J-W, Bartel DP (2015). Predicting effective microRNA target sites in mammalian mRNAs. Elife.

[36] Naldi A, Hernandez C, Abou-Jaoudé W, Monteiro PT, Chaouiya C, Thieffry D (2018). Logical modeling and analysis of cellular regulatory networks with GINsim 3.0. Front Physiol.

[37] Abou-Jaoudé W, Traynard P, Monteiro PT, Saez-Rodriguez J, Helikar T, Thieffry D, Chaouiya C (2016). Logical modeling and dynamical analysis of cellular networks. Front. Genet..

[38] Silveira DA, Gupta S, Mombach JCM (2020). Systems biology approach suggests new miRNAs as phenotypic stability factors in the epithelial-mesenchymal transition. J. R. Soc. Interface.

[39] Xie X, Le L, Fan Y, Lv L, Zhang J (2012). Autophagy is induced through the ROS-TP53-DRAM1 pathway in response to mitochondrial protein synthesis inhibition. Autophagy.

[40] Patel N, Garikapati KR, Pandita RK, Singh DK, Pandita TK, Bhadra U, Bhadra MP (2017). miR-15a/miR-16 down-regulates BMI1, impacting Ub-H2A mediated DNA repair and breast cancer cell sensitivity to doxorubicin. Sci. Rep..

[41] Fernandez A, O’Leary C, O’Byrne KJ, Burgess J, Richard DJ, Suraweera A (2021). Epigenetic mechanisms in DNA double strand break repair: A clinical review. Front. Mol. Biosci..

[42] Salzman DW, Nakamura K, Nallur S, Dookwah MT, Metheetrairut C, Slack FJ, Weidhaas JB (2016). miR-34 activity is modulated through 5′-end phosphorylation in response to DNA damage. Nat. Commun..

[43] Suzuki HI, Yamagata K, Sugimoto K, Iwamoto T, Kato S, Miyazono K (2009). Modulation of microRNA processing by p53. Nature.

[44] Bar-Or RL, Maya R, Segel LA, Alon U, Levine AJ, Oren M (2000). Generation of oscillations by the p53-Mdm2 feedback loop: A theoretical and experimental study. Proc. Natl. Acad. Sci..

[45] Mao A, Zhao Q, Zhou X, Sun C, Si J, Zhou R, Gan L, Zhang H (2016). MicroRNA-449a enhances radiosensitivity by downregulation of c-Myc in prostate cancer cells. Sci. Rep..

[46] Gupta S, Silveira DA, Mombach JCM (2020). Towards DNA-damage induced autophagy: A Boolean model of p53-induced cell fate mechanisms. DNA Repair.

[47] Gupta S, Silveira DA, Mombach JCM (2018). Modeling the role of microRNA-449a in the regulation of the G2/M cell cycle checkpoint in prostate LNCaP cells under ionizing radiation. PLoS ONE.

[48] Gupta S, Silveira DA, Mombach JCM (2020). ATM/miR-34a-5p axis regulates a p21-dependent senescence-apoptosis switch in non-small cell lung cancer: A Boolean model of G1/S checkpoint regulation. FEBS Lett..

[49] Silveira, D. A., Gupta, S. & Mombach, J. C. P53/E2F1/miR-25 axis regulates apoptosis induction in glioblastoma cells: A qualitative model. *J. Phys. Complex* (2020).

[50] Gupta S, Silveira DA, Barbé-Tuana FM, Mombach JCM (2020). Integrative data modeling from lung and lymphatic cancer predicts functional roles for miR-34a and miR-16 in cell fate regulation. Sci. Rep..

[51] Gupta S, Hashimoto RF (2022). Dynamical analysis of a boolean network model of the Oncogene role of lncRNA ANRIL and lncRNA UFC1 in non-small cell lung cancer. Biomolecules.

[52] Carnero, A. Markers of cellular senescence. In *Cell senescence* pp. 63–81. Springer (2013). 10.1007/978-1-62703-239-1_4 23296651

[53] Kim YC, Guan K-L (2015). mTOR: A pharmacologic target for autophagy regulation. J. Clin. Investig..

[54] Alers S, Löffler AS, Wesselborg S, Stork B (2012). Role of AMPK-mTOR-Ulk1/2 in the regulation of autophagy: Cross talk, shortcuts, and feedbacks. Mol. Cell. Biol..

[55] Nazio F, Strappazzon F, Antonioli M, Bielli P, Cianfanelli V, Bordi M, Gretzmeier C, Dengjel J, Piacentini M, Fimia GM (2013). mTOR inhibits autophagy by controlling ULK1 ubiquitylation, self-association and function through AMBRA1 and TRAF6. Nat. Cell Biol..

[56] Crighton D, Wilkinson S, O’Prey J, Syed N, Smith P, Harrison PR, Gasco M, Garrone O, Crook T, Ryan KM (2006). DRAM, a p53-induced modulator of autophagy, is critical for apoptosis. Cell.

[57] Lu T, Zhu Z, Wu J, She H, Han R, Xu H, Qin Z-H (2019). DRAM1 regulates autophagy and cell proliferation via inhibition of the phosphoinositide 3-kinase-Akt-mTOR-ribosomal protein S6 pathway. Cell Commun. Signal..

[58] Bertoli C, Skotheim JM, De Bruin RA (2013). Control of cell cycle transcription during G1 and S phases. Nat. Rev. Mol. Cell Biol..

[59] Mendes ND, Henriques R, Remy E, Carneiro J, Monteiro PT, Chaouiya C (2018). Estimating attractor reachability in asynchronous logical models. Front. Physiol..

[60] Singh Y, Garden OA, Lang F, Cobb BS (2015). MicroRNA-15b/16 enhances the induction of regulatory T cells by regulating the expression of rictor and mTOR. J. Immunol..

[61] Kitadate A, Ikeda S, Teshima K, Ito M, Toyota I, Hasunuma N, Takahashi N, Miyagaki T, Sugaya M, Tagawa H (2016). MicroRNA-16 mediates the regulation of a senescence–apoptosis switch in cutaneous T-cell and other non-Hodgkin lymphomas. Oncogene.

[62] Cimmino A, Calin GA, Fabbri M, Iorio MV, Ferracin M, Shimizu M, Wojcik SE, Aqeilan RI, Zupo S, Dono M (2005). miR-15 and miR-16 induce apoptosis by targeting BCL2. Proc. Natl. Acad. Sci..

[63] Zajkowicz A, Gdowicz-Kłosok A, Krześniak M, Ścieglińska D, Rusin M (2015). Actinomycin D and nutlin-3a synergistically promote phosphorylation of p53 on serine 46 in cancer cell lines of different origin. Cell Signal.

[64] Sun F, Fu H, Liu Q, Tie Y, Zhu J, Xing R, Sun Z, Zheng X (2008). Downregulation of CCND1 and CDK6 by miR-34a induces cell cycle arrest. FEBS Lett..

[65] He X, Yang A, McDonald DG, Riemer EC, Vanek KN, Schulte BA, Wang GY (2017). MiR-34a modulates ionizing radiation-induced senescence in lung cancer cells. Oncotarget.

[66] Hermeking H (2010). The miR-34 family in cancer and apoptosis. Cell Death Differ.

[67] Feliciano A, Sánchez-Sendra B, Kondoh H, LLeonart ME,  (2011). MicroRNAs regulate key effector pathways of senescence. J. Aging Res..

[68] Wei B, Song Y, Zhang Y, Hu M (2013). microRNA-449a functions as a tumor-suppressor in gastric adenocarcinoma by targeting Bcl-2. Oncol. Lett..

