# Supplementary material for: Dynamical modeling of miR-34a, miR-449a, and miR-16 reveals numerous DDR signaling pathways regulating senescence, autophagy, and apoptosis in HeLa cells
Source: Sci Rep. 2022 Mar 22;12:4911. doi: 10.1038/s41598-022-08900-y (PMC8941124; doi:10.1038/s41598-022-08900-y)
Supplement: Supplementary file 1 — Supplementary Information 1. [file 41598_2022_8900_MOESM1_ESM.docx]

**Dynamical modeling of miR-34a, miR-449a, and miR-16 reveals numerous DDR signaling pathways regulating senescence, autophagy, and apoptosis in HeLa cells**

**Author Information**

Shantanu Gupta^1*‡^, Pritam Kumar Panda^2‡^, Ronaldo F. Hashimoto^1,‡^, Shailesh Kumar Samal^3^, Suman Mishra^4^, Suresh Kr. Verma^2^, Yogendra Kumar Mishra^5^, Rajeev Ahuja^*2^

Affiliations

**^1^ Instituto de Matemática e Estatística, Departamento de Ciência da Computação, Universidade de São Paulo, Rua do Matão 1010, 05508-090, São Paulo - SP, Brasil**

Shantanu Gupta, Ronaldo F. Hashimoto

**^2^Condensed Matter Theory Group, Materials Theory Division, Department of Physics and Astronomy, Uppsala University, Box 516, SE-751 20 Uppsala, Sweden**

Pritam Kumar Panda, Suresh Kr. Verma and Rajeev Ahuja

**^3^Unit of Immunology and Chronic Disease, Institute of Environmental Medicine, Karolinska Institutet, 17177 Stockholm, Sweden**

Shailesh Samal

**^4^School of Biotechnology, KIIT University, Bhubaneswar, 751024**

Suman Mishra

**^5^Mads Clausen Institute, NanoSYD, University of Southern Denmark, Alsion 2, DK-6400 Sønderborg, Denmark**

Yogendra Kumar Mishra

Contributions

S.G, and P.K.P designed and conceptualized the study, S.G, P.K.P and R.F.H analyzed the results and wrote the manuscript; P.K.P, S.M designed all the illustrations; S.G. verified and provided feedback to the illustrations; S.K.S, S.M, S.K.V, R.A and Y.K.M provided critical feedback and edited the manuscript. All authors approved the final version of the manuscript.

^‡^Equally contributing authors

Corresponding author

^*^Correspondence to: Shantanu Gupta (S.G) and Rajeev Ahuja (R.A): Email: [shantanubrasil1@gmail.com](mailto:shantanubrasil1@gmail.com)

& [rajeev.ahuja@physics.uu.se](mailto:rajeev.ahuja@physics.uu.se)

Co-authors: P.K.P: [pritam.panda@physics.uu.se](mailto:pritam.panda@physics.uu.se) R.F.H: [ronaldo@ime.usp.br](mailto:ronaldo@ime.usp.br) S.K.S: [shailesh.samal@ki.se](mailto:shailesh.samal@ki.se) S.M: [sumanmishra2615@gmail.com](mailto:sumanmishra2615@gmail.com) S.K.V: [suresh.verma@physics.uu.se](mailto:suresh.verma@physics.uu.se) Y.K.M: [mishra@mci.sdu.dk](mailto:mishra@mci.sdu.dk) R.A: [rajeev.ahuja@physics.uu.se](mailto:rajeev.ahuja@physics.uu.se)

**Supplementary Table S1**

**Table S1:** Logical rules that control node states in the model (Fig 2). The logical operators AND, OR and NOT are used to define the rules for each node in terms of the state of its regulators. Left-hand side lists the official names of the molecules (target node). The right-hand side lists the references corresponding to each upcoming interaction of the target node.

| **Oﬃcial names of the molecules** | **Target node** | **Interactions** | **Descriptions** | **References (PubMed IDs)** |
| --- | --- | --- | --- | --- |
|  |  |  |  |  |
| **microRNA-16** | miR-16 | Transfected_miRNA | Transfected_miRNA activate miR-16 | PMID: 25945419 |
|  |  | ATM | ATM can activate miR-16 | PMID: 20668064 |
|  |  | Rule: ATM ***AND*** Transfected_miRNA | | miR-16 can be activated in the presence of ATM AND Transfected_miRNA. |
|  |  |  |  |  |
|  |  |  |  |  |
| **microRNA-34a** | miR-34a | Transfected_miRNA | Transfected_miRNA activate miR-34a | PMID: 33028635 |
|  |  | ATM | ATM can activate miR-34a | PMID: 26996824 |
|  |  | Rule: ATM ***AND*** Transfected_miRNA | | miR-34a can be activated in the presence of ATM AND Transfected_miRNA. |
|  |  |  |  |  |
|  |  |  |  |  |
| **microRNA-449a** | miR-449a | Transfected_miRNA | Transfected_miRNA activate miR-449a | PMID: 33028635 |
|  |  | DDR_Activation | DNA damage can activates miR-449a | PMID: 27250340 |
|  |  | E2F1 | E2F1 can activates miR-449a | PMID: 19960022 |
|  |  | Rule: (E2F1 ***OR*** DDR_Activation) ***AND*** Transfected_miRNA | | miR-449a can be activated by E2F1 OR DNA damage AND transfection of miR-449a |
|  |  |  |  |  |
|  |  |  |  |  |
| **Protein Nef** | HIV1_Nef | miR-16 | miR-16 inhibits HIV1_Nef | PMID: 24678387 |
|  |  | Transfected_miRNA | Transfected_miRNA inhibist HIV1_Nef | PMID: 24678387 |
|  |  | DDR_Activation | DDR_Activation inhibits HIV1_Nef | PMID: 28388353 |
|  |  | Rule: ***NOT*** (miR16 ***OR*** Transfected_miRNA ***OR*** DDR_Activation) | | HIV1_Nef can be activated in the absence of miR-16 OR in the absence of DNA damage |
|  |  |  |  |  |
|  |  |  |  |  |
| **Phosphofurin acidic cluster sorting protein 1** | PACS1 | miR-34a | PACS1 is direct target of miR-34a | PMID: 33028635 |
|  |  | miR-449a | PACS1 is direct target of miR-449a | PMID: 33028635 |
|  |  | DDR_Activation | DDR_Activation inhibits PACS1 | PMID: 33028635 |
|  |  | HIV1_Nef | HIV1_Nef activates PACS1 | PMID: 10707087 |
|  |  | Rule: ***NOT*** (miR34a ***OR*** miR449a ***OR*** DDR_Activation) ***AND*** HIV1_Nef | | PACS1 can be activated in the absence of miR-34a OR in the absence of miR-449a OR in the absence of DNA damage AND in the presence of HIV1_Nef |
|  |  |  |  |  |
|  |  |  |  |  |
|  |  |  |  |  |
|  |  |  |  |  |
| **Human Papillomavirus E6** | HVPE6 | DDR_Activation | DNA Damage inhibits E6 | PMID: 6690061 |
|  |  | Rule: ***NOT*** DDR_Activation | | DNA Damage inhibits HVPE6 |
|  |  |  |  |  |
| **DNA Damage response activation** | DDR_Activation | Cdc25A | Suppression of Cdc25A, intensify DDR_Activation | PMID: 19536137 |
|  |  | PACS1 | Targeting PACS1 increased DDR activation | PMID: 33028635 |
|  |  | Wip1 | Repression of Wip1, enhanced the activation of DDR | PMID: 28903382 |
|  |  | BMI1 | Repression of BMI, escalate the activation of DDR | PMID: 28655885 |
|  |  | Transfected_miRNA | Transfected_miRNA increase DDR_Activation | PMID: 19536137 |
|  |  | Rule: Transfected_miRNA ***AND NOT*** (Wip1 **AND** BMI1) | | DDR activation, in the presence of Transfected_miRNA AND in the absence of Cdc25A AND Wip1 AND BMI1. |
|  |  |  |  |  |
|  |  |  |  |  |
|  |  |  |  |  |
| **ATM serine/threonine kinase** | ATM | Wip1 | ATM suppressed by Wip1 | PMID: 16949371 |
|  |  | HVPE6 | HVPE6 inhibits ATM expression | PMID: 23706308 |
|  |  | DDR_Activation | DDR_Activation directly activate ATM | PMID: 24003211 |
|  |  | Rule: DDR_Activation ***AND NOT*** Wip1 | | ATM activated, In the presence of DDR AND in the absence of Wip1. |
|  |  |  |  |  |
| **Mitogen-activated protein kinase 1** | MAPK | DDR_Activation | DDR_Activation can activate AMPK | PMID: 12970909 |
|  |  | ATM | ATM can activate AMPK | PMID: 11682011 |
|  |  | ULK1 | ULK1 directly target AMPK activity | PMID: 29732413 |
|  |  | Rule: (ATM ***OR*** DDR_Activation) ***AND NOT*** ULK1 | | MAPK activated, In the presence of ATM OR DDR activation AND In the absence of ULK1. |
|  |  |  |  |  |
|  |  |  |  |  |
| **AMP activated protein kinase** | AMPK | ATM | ATM can activate AMPK | PMID: 24737504 |
|  |  | DDR_Activation | DDR_Activation can activate AMPK | PMID: 24100703 |
|  |  | ULK1 | ULK1 directly target AMPK activity | PMID: 27557493 |
|  |  | Rule: (ATM ***OR*** DDR_Activation) ***AND NOT*** ULK1 | | AMPK activated, In the presence of ATM OR DDR activation AND In the absence of ULK1. |
|  |  |  |  |  |
|  |  |  |  |  |
| **RAC-alpha serine/threonine-protein kinase** | AKT | DDR_Activation | DDR_Activation inhibits AKT expression | PMID: 22607554 |
|  |  | PTEN | PTEN directly target AKT | PMID: 21779440 |
|  |  | mTORC2 | mTORC2 activates AKT | PMID: 26235620 |
|  |  | BMI1 | BMI1 activates AKT expression | PMID: 23092893 |
|  |  | Rule: ***NOT*** (DDR_Activation ***OR*** PTEN) ***OR*** mTORC2 ***OR*** BMI1 | | Activation of AKT, In the absence of DDR_Activation AND PTEN OR In the presence of mTORC2 AND BMI1. |
|  |  |  |  |  |
|  |  |  |  |  |
| **Tumor supressor p53 protein** | Tumor supressor p53 protein (Ser-15 and Ser-20) **p53_A** | Sirt1 | Sirt1 inhibits p53 activity | PMID: 30739913 |
|  |  | p53_K | p53_A suppressed by p53_K | PMID: 21576488 |
|  |  | ATM | ATM can activates p53_A expression | PMID: 11526498 |
|  |  | p53INP1 | Control of p53 accumulation | PMID: 21576488 |
|  |  | MAPK | MAPK can activates p53_A | PMID: 14764989 |
|  |  | AMPK | AMPK can trigger p53_A expression | PMID: 22728651 |
|  |  | Wip1 | p53_A suppressed by Wip1 | PMID: 17936559 |
|  |  | Mdm2 | Mdm2 inhibits p53_A expression | PMID: 14707283 |
|  |  | HVPE6 | HVPE6 inhibits p53_A expression | PMID: 22244155 |
|  |  | Rule: ***NOT*** Sirt1 AND ***NOT*** p53_K ***AND*** (ATM ***OR NOT*** p53INP1 ***OR*** (MAPK ***AND*** AMPK ***AND NOT*** Wip1 ***AND NOT*** Mdm2 ***AND NOT*** HVPE6)) | | p53-A can be activated in the absence of Sirt1 and in the absence of p53-K and in the presence of ATM OR in the absence p53-INP1 or in the presence of MAPK AND in the presence of AMPK AND In the absence of Wip1 AND Mdm2 AND HVPE6. |
|  |  |  |  |  |
|  |  |  |  |  |
|  |  |  |  |  |
|  |  |  |  |  |
|  |  |  |  |  |
|  |  |  |  |  |
|  | Tumor supressor p53 protein (Ser-46) **p53_K** | p53_A | p53_A inhibits p53_K | PMID: 21576488 |
|  |  | Sirt1 | Sirt1 inhibits p53 | PMID: 30739913 |
|  |  | Wip1 | Wip1 inhibits p53 | PMID: 17936559 |
|  |  | ATM | ATM can activates p53_K expression | PMID: 11526498 |
|  |  | Mdm2 | Mdm2 inhibits p53_K expression | PMID: 14707283 |
|  |  | HVPE6 | HVPE6 inhibits p53_K expression | PMID: 22244155 |
|  |  | Rule: ***NOT*** p53_A ***AND*** (***NOT*** Sirt1 ***OR NOT*** Wip1) ***AND*** ATM ***AND NOT*** Mdm2 ***AND NOT*** HVPE6 | | p53-K can be activated in the absence of p53-A and in the absence of Sirt1 or in the absence of Wip1 and in the presence of ATM AND in the absence of Mdm2 AND HVPE6 |
|  |  |  |  |  |
|  |  |  |  |  |
|  |  |  |  |  |
|  |  |  |  |  |
| **Tumor protein p53 inducible nuclear protein 1** | p53INP1 | p53_A | p53 Ser-15 and Ser-20 activate p53-INP1 | PMID: 23717325 |
|  |  | p53_K | p53 Ser-46 activates p53-INP1 | PMID: 11030628 |
|  |  | Rule: p53_A ***OR*** p53_K | | p53INP1 activation: In the presence of p53-A OR In the presence of p53-K. |
|  |  |  |  |  |
| **Sirtuin 1** | Sirt1 | E2F1 | E2F1 activates Sirt1 | PMID: 16892051 |
|  |  | miR-34a | Sirt1 is direct target of miR-34a | PMID: 25826085 |
|  |  | miR-449a | Sirt1 is direct target of miR-449a | PMID: 21418558 |
|  |  | Rule: E2F1 ***AND NOT*** miR34a ***AND NOT*** miR449a | | Sirt1 can be activated in the presence of E2F1 AND in the absence of miR-34a AND in the absence of miR-449a |
|  |  |  |  |  |
|  |  |  |  |  |
| **E3 ubiquitin protein ligase homolog protein** | Mdm2 | Wip1 | Wip1 negatively regulates Mdm2 | PMID: 17936559 |
|  |  | p53_A | p53 activates Mdm2 | PMID: 14707283 |
|  |  | ATM | ATM inhibits Mdm2 | PMID: 16082221 |
|  |  | AKT | AKT activate Mdm2 | PMID: 11923280 |
|  |  | Rule: ***(NOT*** Wip1 ***OR*** p53_A) ***AND NOT*** ATM ***AND*** AKT | | Mdm2 activated in the presence of p53 OR in the absence of Wip1 AND in the absence of ATM AND In the presence of AKT. |
|  |  |  |  |  |
|  |  |  |  |  |
|  |  |  |  |  |
| **Myc proto-oncogene protein** | Myc | E2F1 | E2F1 directly activates c-Myc | PMID: 18345030 |
|  |  | MAPK | MAPK increase Myc activity | PMID: 16365184 |
|  |  | RB | RB directly inhibits Myc expression | PMID: 27105536 |
|  |  | p21 | p21 directly inhibits Myc | PMID: 11274368 |
|  |  | miR-34a | Myc is direct target of miR-34a | PMID: 25686834 |
|  |  | miR-449a | Myc is direct target of miR-449a | PMID: 27250340 |
|  |  | Rule: (E2F1 ***OR*** MAPK) ***AND NOT*** RB ***AND NOT*** p21 ***AND NOT*** miR34a ***AND NOT*** miR449a | | Myc activation: In the presence of E2F1 OR AMPK AND In the absence of RB AND p21 AND miR-34a AND miR-449a |
|  |  |  |  |  |
|  |  |  |  |  |
|  |  |  |  |  |
| **Cyclin-dependent kinase inhibitor 1A** | p21 | p53_A | p53 activates p21 | PMID: 28288132 |
|  |  | Myc | Repression of c-Myc activates p21 expression | PMID: 12384701 |
|  |  | Caspase3 | Caspase3 inhibits p21 | PMID: 10022118 |
|  |  | BMI1 | Repression of BMI-1 activates p21 expression | PMID: 26640145 |
|  |  | AKT | AKT inhibits p21 expression | PMID: 16443763 |
|  |  | Rule: p53_A ***OR*** (***NOT*** Myc ***AND NOT*** Caspase3 ***AND NOT*** BMI1 ***AND NOT*** AKT) | | p21 can be activated in the presence of p53-A AND In the absence of Caspase3 AND In the absence of BMI1 AND In the absence of AKT. |
|  |  |  |  |  |
|  |  |  |  |  |
|  |  |  |  |  |
| **DNA damage-regulated autophagy modulator protein 1** | DRAM1 | p53_K | DRAM1 is activated by p53-K | PMID: 25989210 |
|  |  | Rule: p53_K | | DRAM1 can be activated in the presence of p53-K. |
|  |  |  |  |  |
| **BCL2 binding component 3** | PUMA | p53_K | PUMA is activated by p53-K | PMID: 11463392 |
|  |  | Rule: p53_K | | PUMA can be activated in the presence of p53-K. |
|  |  |  |  |  |
| **Mg2+/Mn2+ dependent 1D** | Wip1 | p53_A | p53_A activates Wip1expression | PMID: 20093361 |
|  |  | miR-16 | miR-16 inhibits Wip1 | PMID: 20668064 |
|  |  | Rule: p53_A ***AND NOT*** miR_16 | | Wip1 expression can be activated in the presence of p53_A AND In the absence of miR-16. |
|  |  |  |  |  |
|  |  |  |  |  |
| **Polycomb complex protein BMI-1** | BMI1 | miR-16 | BMI1 is a direct target of miR-16 | PMID: 19903841 |
|  |  | E2F1 | E2F1 can activate BMI1 expression | PMID: 16582100 |
|  |  | Myc | Myc activates BMI1 expression | PMID: 23239878 |
|  |  | Rule: ***NOT*** miR_16 AND (E2F1 ***OR*** Myc) | | BMI1 can be activated in the absence of miR-16 AND In the presence of E2F1 OR In the presence of Myc. |
|  |  |  |  |  |
|  |  |  |  |  |
| **Target of rapamycin complex 2 subunit MAPKAP1** | mTORC2 | miR-16 | mTORC2 is a direct target of miR-16 | PMID: 25945419 |
|  |  | miR-34a | mTORC2 is a direct target of miR-34a | PMID: 24944883 |
|  |  | AKT | AKT activates mTORC2 | PMID: 26235620 |
|  |  | DRAM1 | mTORC2 is inhibited by DRAM1 | PMID: 22525272 |
|  |  | mTORC1 | mTORC1 inhibits mTORC2 activity | PMID: 29232655 |
|  |  | Sirt1 | Sirt1 inhibits mTORC2 activity | PMID: 21965330 |
|  |  | AMPK | mTORC2 is a direct target of AMPK | PMID: 19625624 |
|  |  | MAPK | mTORC2 is a direct target of MAPK | PMID: 27990160 |
|  |  | Rule: (***NOT*** miR-34a ***AND*** ***NOT*** miR_16) ***OR*** AKT ***OR NOT*** (DRAM1 ***OR*** mTORC1 ***OR*** Sirt1 ***OR*** AMPK ***OR*** MAPK) | | mTORC2 can be activated,In the absence of miR-16 AND in the absence of miR-34a OR in the presence of AKT OR In the absence of DRAM1 OR mTORC1 OR Sirt1 OR AMPK OR MAPK. |
|  |  |  |  |  |
|  |  |  |  |  |
|  |  |  |  |  |
|  |  |  |  |  |
|  |  |  |  |  |
| **Serine/threonine-protein kinase mTOR** | mTORC1 | AKT | mTORC1 activated by AKT | PMID: 16027121 |
|  |  | ULK1 | Repression of ULK1 activates mTORC1 expression | PMID: 21795849 |
|  |  | DRAM1 | DRAM1 inhibits mTORC1 expression | PMID: 30902093 |
|  |  | miR-16 | mTORC1 is directly target of miR-16 | PMID: 26538392 |
|  |  | AMPK | mTORC1 is a direct target of AMPK | PMID: 22025673 |
|  |  | MAPK | mTORC1 is a direct target of MAPK | PMID: 18725988 |
|  |  | Rule: AKT ***AND NOT*** (ULK1 ***AND*** DRAM1 ***AND*** miR_16 ***OR*** AMPK ***OR*** MAPK) | | mTORC1 can be activated, In the presence of AKT AND in the absence of ULK1 AND DRAM1 AND miR-16 OR AMPK OR MAPK. |
|  |  |  |  |  |
|  |  |  |  |  |
|  |  |  |  |  |
| **E2F transcripition factor 1** | E2F1 | RB | RB inhibits E2F1 expression | PMID: 9315635 |
|  |  | Cdc25A | Cdc25A can activates E2F1 expression | PMID: 10454584 |
|  |  | ATM | ATM can activates E2F1 | PMID: 18235226 |
|  |  | Sirt1 | Sirt1 inhibits E2F1 | PMID: 16892051 |
|  |  | Myc | Myc activates E2F1 expression | PMID: 15944709 |
|  |  | miR-34a | E2F1 is direct target of miR-34a | PMID: 29036883 |
|  |  | miR-449a | E2F1 is direct target of miR-449a | PMID: 19833767 |
|  |  | Rule: (***NOT*** RB AND ((Cdc25A ***AND*** ATM) ***OR NOT*** Sirt1 ***OR NOT*** miR-34a ***OR NOT*** miR-449a)) ***OR*** Myc | | E2F1 can be activated in the absence of RB AND in the presence of Cdc25A AND in the presence of ATM OR in the absence of Sirt1 OR NOT in the absence of miR-34a OR NOT in the absence of miR-449a OR in the presence of c-Myc. |
|  |  |  |  |  |
|  |  |  |  |  |
|  |  |  |  |  |
|  |  |  |  |  |
|  |  |  |  |  |
|  |  |  |  |  |
| **Retinoblastoma 1 protein** | RB | CDK46_CycD | CDK46-CycD directly inhibits RB | PMID: 30061045 |
|  |  | CDK2_CycE | CDK2-CycE directly inhibits RB | PMID: 23877564 |
|  |  | HVPE6 | HVPE6 inhibits RB expression | PMID: 19721808 |
|  |  | Rule: (***NOT*** CDK46_CycD ***AND NOT*** CDK2_CycE) ***OR NOT*** HVPE6 | | RB can be activated in the absence of CDK46_CycD AND in the absence of CDK2_CycE OR in the absence of HVPE6 |
|  |  |  |  |  |
|  |  |  |  |  |
|  |  |  |  |  |
| **Cyclin-dependent kinases 4 and 6 complex/CyclinD1** | CDK46_CycD | Cdc25A | Cdc25A activates CDK46-CycD | PMID: 28192398 |
|  |  | miR-16 | CDK46-CycD is a direct target of miR-16 | PMID: 18701644 |
|  |  | miR-34a | CDK46-CycD is a direct target of miR-34a | PMID: 18406353 |
|  |  | miR-449a | CDK46-CycD is a direct target of miR-449a | PMID: 24993091 |
|  |  | p21 | p21 directly inhibits CDK46-CycD | PMID: 26658964 |
|  |  | Rule: Cdc25A ***AND NOT*** miR_16 ***AND NOT*** p21 ***AND NOT*** miR-34a ***AND NOT*** miR-449a | | CDK46_CycD can be activated in the presence of Cdc25A AND in the absence of miR-16 AND in the absence of p21 AND in the absence of miR-34a AND in the absence of miR-449a |
|  |  |  |  |  |
|  |  |  |  |  |
|  |  |  |  |  |
|  |  |  |  |  |
|  |  |  |  |  |
| **Cyclin-dependent kinase 2/CyclinE2** | CDK2_CycE | Cdc25A | Cdc25A activates CDK2-CycE | PMID: 12801928 |
|  |  | E2F1 | E2F1 activates CDK2-CycE | PMID: 1388288 |
|  |  | miR-16 | CDK2-CycE is a direct target of miR-16 | PMID: 18701644 |
|  |  | miR-34a | CDK2-CycE is a direct target of miR-34a | PMID: 18406353 |
|  |  | miR-449a | CDK2-CycE is a direct target of miR-449a | PMID: 24993091 |
|  |  | p21 | p21 directly inhibits CDK2-CycE | PMID: 9548727 |
|  |  | Rule: Cdc25A ***AND*** E2F1 ***AND*** NOT miR_16 ***AND NOT*** p21 ***AND NOT*** miR-34a ***AND NOT*** miR-449a | | CDK2_CycE can be activated in the presence of Cdc25A AND in the presence of E2F1 AND in the absence of miR-16 AND in the absence of p21 in the absence of miR-34a AND in the absence of miR-449a. |
|  |  |  |  |  |
|  |  |  |  |  |
|  |  |  |  |  |
|  |  |  |  |  |
|  |  |  |  |  |
| **Cell division cycle 25A** | Cdc25A | ATM | ATM directly inhibits Cdc25A | PMID: 23508805 |
|  |  | miR-16 | Cdc25A is a direct target of miR-16 | PMID: 19536137 |
|  |  | miR-34a | Cdc25A is a direct target of miR-34a | PMID: 18406353 |
|  |  | miR-449a | Cdc25A is a direct target of miR-449a | PMID: 24993091 |
|  |  | Rule: ***NOT*** ATM ***AND NOT*** miR_16 ***AND NOT*** miR-34a ***AND NOT*** miR-449a | | Cdc25A can be activated in the absence of ATM AND in the absence of miR-16 AND in the absence of miR-34a AND in the absence of miR-449a |
|  |  |  |  |  |
|  |  |  |  |  |
|  |  |  |  |  |
| **Caspase-3** | Caspase3 | BCL2 | BCL2 inhibits Caspase3 | PMID: 10409669 |
|  |  | p21 | p21 inhibits Caspase3 | PMID: 10884382 |
|  |  | BAX | BAX enhances Caspase3 activity | PMID: 30485804 |
|  |  | Rule: BAX ***OR*** ***(NOT*** p21 ***AND*** ***NOT*** BCL2) | | Caspase3 can be activated in the absence of BCL2 AND in the absence of p21 OR in the presence of BAX. |
|  |  |  |  |  |
|  |  |  |  |  |
| **BCL2 associated X, apoptosis regulator** | BAX | BCL2 | BCL2 directly inhibits BAX | PMID: 10713725 |
|  |  | DRAM1 | DRAM1 can activates BAX expression | PMID: 25633293 |
|  |  | Wip1 | Wip1 inhibits BAX | PMID: 23907458 |
|  |  | Rule: ***NOT*** BCL2 ***AND*** DRAM1 ***AND NOT*** Wip1 | | BAX can be activated in the absence of BCL2 AND in the presence of DRAM1 OR in the absence of Wip1. |
|  |  |  |  |  |
|  |  |  |  |  |
| **BCL2 apoptosis regulator** | BCL2 | miR-16 | BCL2 is a direct target of miR-16 | PMID: 16166262 |
|  |  | PUMA | PUMA inhibits BCL2 expression | PMID: 19641508 |
|  |  | DRAM1 | DRAM1 inhibits BCL2 expression | PMID: 29497611 |
|  |  | miR-34a | BCL2 is a direct target of miR-34a | PMID: 30221494 |
|  |  | miR-449a | BCL2 is a direct target of miR-499a | PMID: 24260067 |
|  |  | MAPK | MAPK directly inhibits BCL2 expression | PMID: 32231094 |
|  |  | Rule: ***NOT*** PUMA ***AND NOT*** DRAM1 ***AND NOT*** MAPK **AND NOT** (miR449a **OR** miR34a **OR** miR16) | | BCL2 can be activated in the absence of PUMA and in the absence of DRAM1 and in the absence of MAPK and in the absence of miR-34a or absence of miR-449a or in the absence of miR-16 |
|  |  |  |  |  |
|  |  |  |  |  |
|  |  |  |  |  |
|  |  |  |  |  |
|  |  |  |  |  |
| **Serine/threonine-protein kinase ULK1** | ULK1 | DRAM1 | DRAM1 activates ULK1 expression | PMID: 30902093 |
|  |  | AMPK | ULK1 can be activated by AMPK | PMID: 22025673 |
|  |  | MAPK | MAPK can activate ULK1 | PMID: 29732413 |
|  |  | mTORC2 | mTORC2 inhibits ULK1 expression | PMID: 20083114 |
|  |  | mTORC1 | mTORC1 inhibits ULK1 expression | PMID: 23524951 |
|  |  | Rule: (DRAM1 ***OR*** MAPK ***OR*** AMPK) ***AND NOT*** mTORC2 ***AND NOT*** mTORC1 | | ULK1 can be activated, In the presence of DRAM1 OR In the presence of AMPK AND in the absence of mTORC2 AND in the absence of mTORC1. |
|  |  |  |  |  |
|  |  |  |  |  |
|  |  |  |  |  |
|  |  |  |  |  |
|  |  |  |  |  |
|  |  |  |  |  |
| **Beclin-1** | Beclin-1 | ATM | ATM can activate Beclin-1 | PMID: 32187724 |
|  |  | BCL2 | BCL2 inhibits Beclin-1 | PMID: 16179260 |
|  |  | ULK1 | ULK1 can activatesBeclin-1 | PMID: 23685627 |
|  |  | Rule: ATM ***AND NOT*** BCL2 ***AND*** ULK1 | | Beclin-1 can be activated in the presence of ATM and in the absence of BCL2 and in the presence of ULK1. |
|  |  |  |  |  |
|  |  |  |  |  |
| **PROLIFERATION** | PROLIFERATION | E2F1 | E2F1 induces Proliferation | PMID: 24023875 |
|  |  | Rule: E2F1 | | PROLIFERATION can be activated in the presence of E2F1. |
|  |  |  |  |  |
|  |  |  |  |  |
| **AUTOPHAGY** | AUTOPHAGY | ULK1 | ULK1 can activates Autophagy | PMID: 23685627 |
|  |  | Rule: ULK1 | | AUTOPHAGY can be activated in the presence of ULK1. |
|  |  |  |  |  |
|  |  |  |  |  |
| **APOPTOSIS** | APOPTOSIS | Caspase3 | Caspase3 is a marker of Apoptosis | PMID: 10200555 |
|  |  | ULK1 | ULK1 suppressed Apoptosis | PMID: 30166400 |
|  |  | DRAM1 | DRAM1 can activates Apoptosis | PMID: 25633293 |
|  |  | Rule: (Caspase3 ***OR*** DRAM1) ***AND NOT*** ULK1 | | APOPTOSIS can be activated in the presence of Caspase3 OR DRAM1 AND in the absence of ULK1. |
|  |  |  |  |  |
|  |  |  |  |  |
| **SENESCENCE** | SENESCENCE | p21 | Senescence induced by p21 | PMID: 21078816 |
|  |  | p53-A | Senescence induced by p53-A | PMID: 23296650 |
|  |  | Rule: p21 ***OR*** p53-A | | SENESCENCE can be activated in the presence of p21 OR in the presence of p53-A. |
|  |  |  |  |  |
|  |  |  |  |  |
