# Supplementary material for: Dynamical modeling of miR-34a, miR-449a, and miR-16 reveals numerous DDR signaling pathways regulating senescence, autophagy, and apoptosis in HeLa cells
Source: Sci Rep. 2022 Mar 22;12:4911. doi: 10.1038/s41598-022-08900-y (PMC8941124; doi:10.1038/s41598-022-08900-y)
Supplement: Supplementary file 2 — Supplementary Information 2. [file 41598_2022_8900_MOESM2_ESM.pdf]

Dynamical modeling of miR-34a, miR-449a, and miR-16 reveals numerous DDR signaling pathways regulating senescence, autophagy, and apoptosis in HeLa cells

Author Information

Shantanu Gupta[1*‡], Pritam Kumar Panda[2‡], Ronaldo F. Hashimoto[1,‡], Shailesh Kumar Samal[3], Suman Mishra[4], Suresh Kr. Verma[2], Yogendra Kumar Mishra[5], Rajeev Ahuja[*2]

Affiliations

[1] Instituto de Matemática e Estatística, Departamento de Ciência da Computação, Universidade de São Paulo, Rua do Matão 1010, 05508-090, São Paulo - SP, Brasil

Shantanu Gupta, Ronaldo F. Hashimoto

[2]Condensed Matter Theory Group, Materials Theory Division, Department of Physics and Astronomy, Uppsala University, Box 516, SE-751 20 Uppsala, Sweden

Pritam Kumar Panda, Suresh Kr. Verma and Rajeev Ahuja

[3]Unit of Immunology and Chronic Disease, Institute of Environmental Medicine, Karolinska Institutet, 17177 Stockholm, Sweden

Shailesh Samal

[4]School of Biotechnology, KIIT University, Bhubaneswar, 751024

Suman Mishra

[5]Mads Clausen Institute, NanoSYD, University of Southern Denmark, Alsion 2, DK-6400 Sønderborg, Denmark

Yogendra Kumar Mishra

[‡]Equally contributing authors

[*]Correspondence to: Shantanu Gupta (S.G) and Rajeev Ahuja (R.A)

Email: shantanubrasil1@gmail.com and rajeev.ahuja@physics.uu.se

Co-authors: P.K.P: pritam.panda@physics.uu.se R.F.H: ronaldo@ime.usp.br S.K.S: shailesh.samal@ki.se S.M: sumanmishra2615@gmail.com S.K.V: suresh.verma@physics.uu.se Y.K.M: mishra@mci.sdu.dk R.A: rajeev.ahuja@physics.uu.se

Supplementary File S1

Agreement between proposed Synthetic Boolean model and experimental data

---

Transfection of miR-16

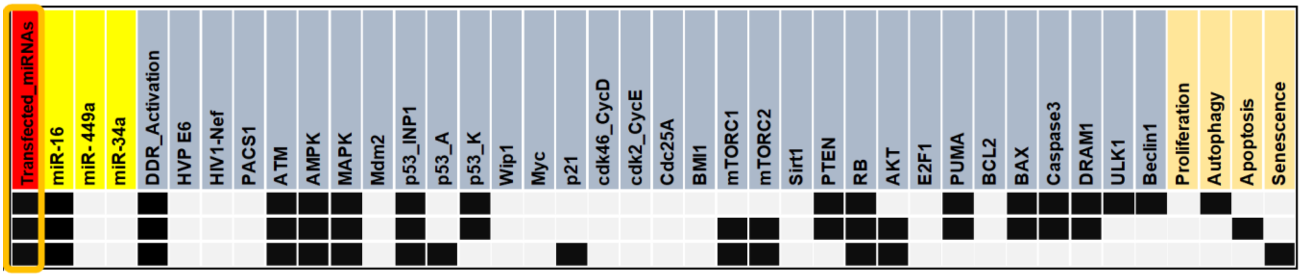

Response/Phenotype: Induction of autophagy, apoptosis and senescence in response to DNA damage

Knockdown (KO) of mTOR2

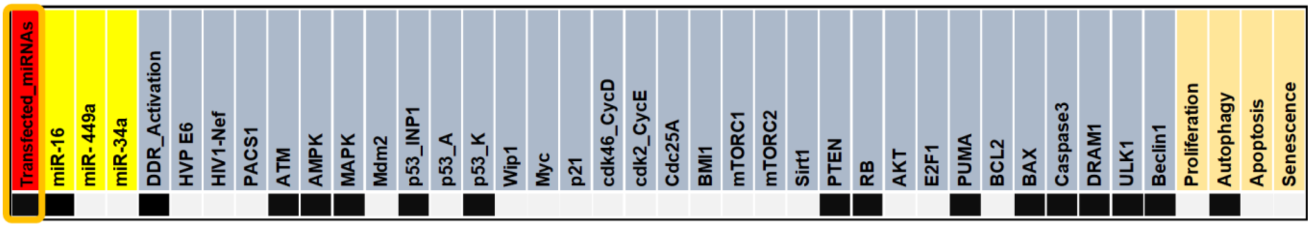

Response/Phenotype: Induction of Autophagy in response to DNA damage

Knockdown (KO) of mTOR1

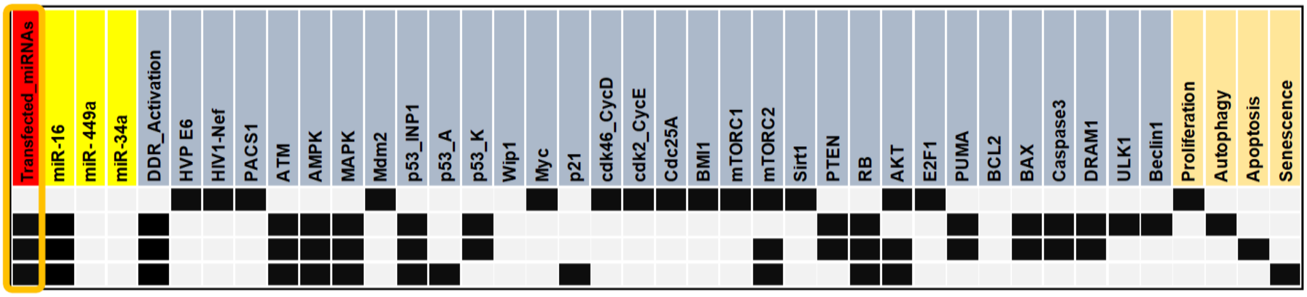

Response/Phenotype: Induction of autophagy, apoptosis and senescence

Transfection of miR-449a

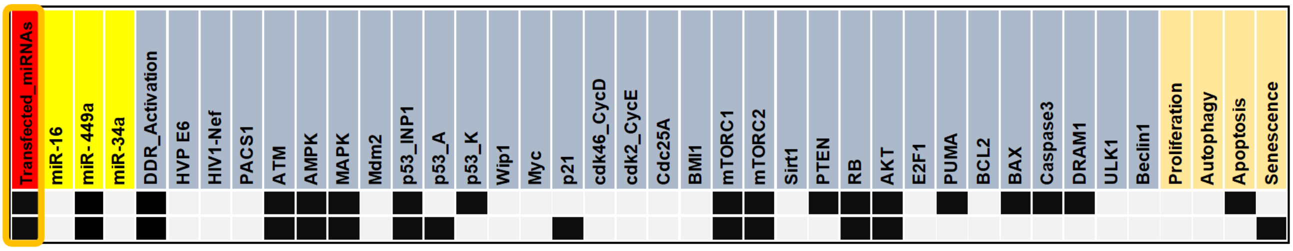

Response/Phenotype: Induction of apoptosis and senescence in response to DNA damage

Transfection of miR-34a

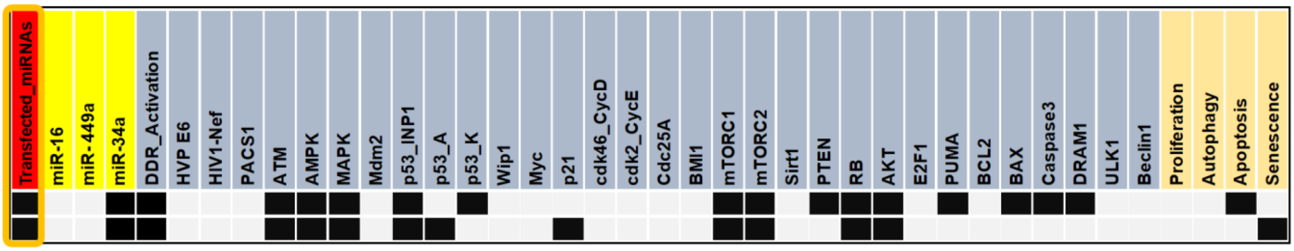

Response/Phenotype: Induction of apoptosis and senescence in response to DNA damage

Knockdown (KO) of PACS1

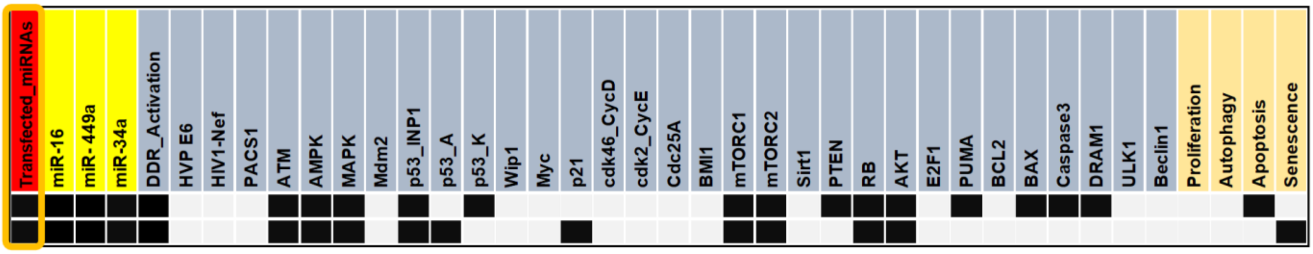

Response/Phenotype: Induction of apoptosis and senescence in response to DNA damage

Synthetic Boolean network predictions based on experimental studies

Synergistic regulation between miR-34a and miR-449a

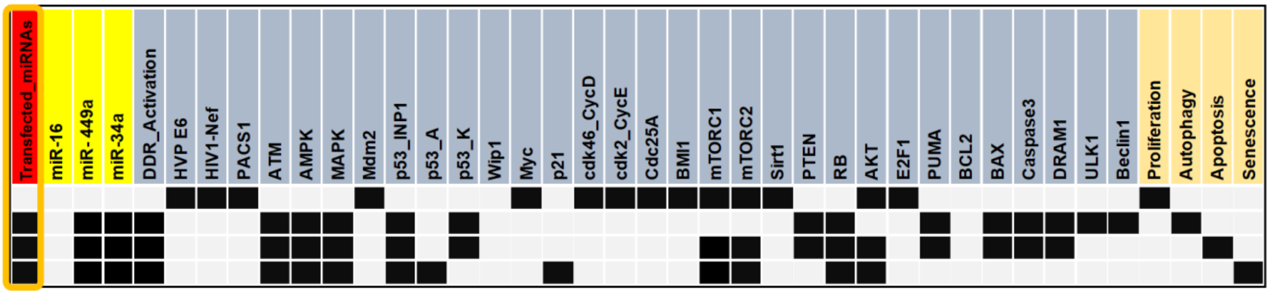

Response/Phenotype: Induction of autophagy, apoptosis and senescence in response to DNA damage

Synergistic regulation between miR-16 and miR-449a

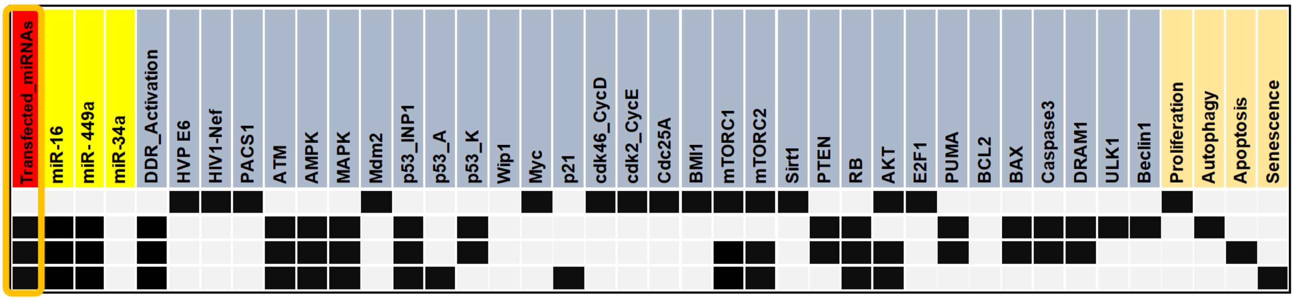

Response/Phenotype: Induction of autophagy, apoptosis and senescence in response to DNA damage

Synergistically overexpression (E1) of miR-449a/miR-34a/miR-16

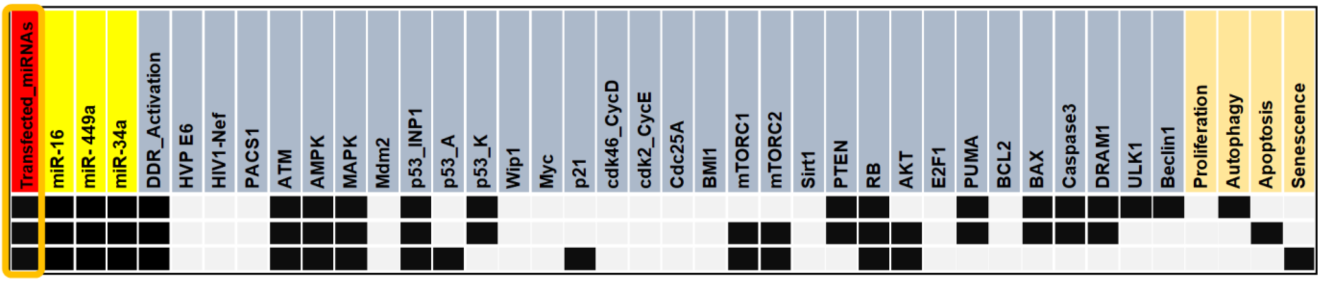

Response/Phenotype: Induction of functional and stable G1/S checkpoint.

Targeting of Cdc25A by miR-16/miR-34a/miR-449a

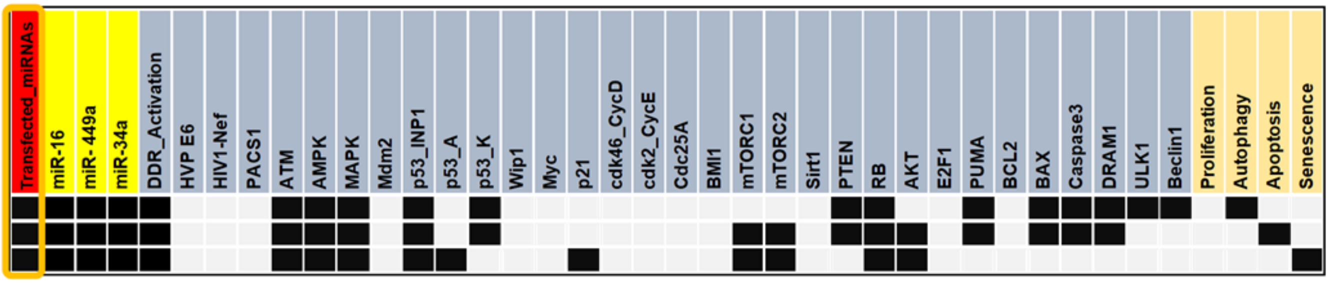

Response/Phenotype: Repression of proliferation through the induction of DNA Damage Response pathways

Knockdown (KO) of PACS1

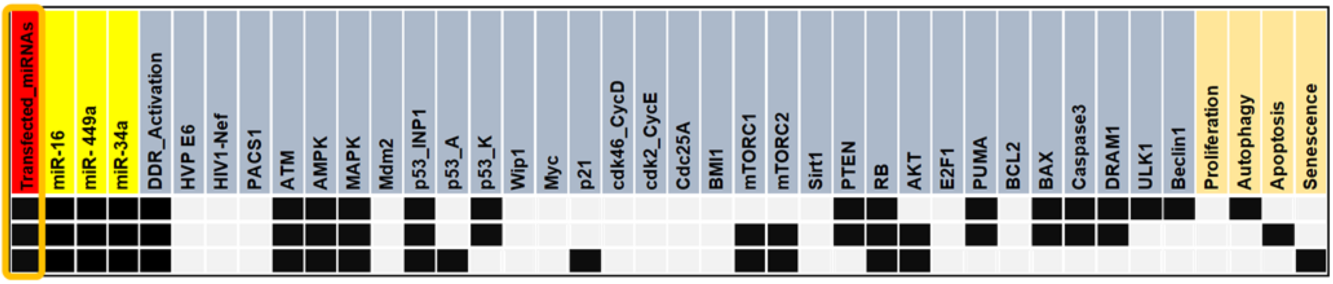

Response/Phenotype: Repression of proliferation through the induction of autophagy along with apoptosis and senescence

Transfection of miR-34a

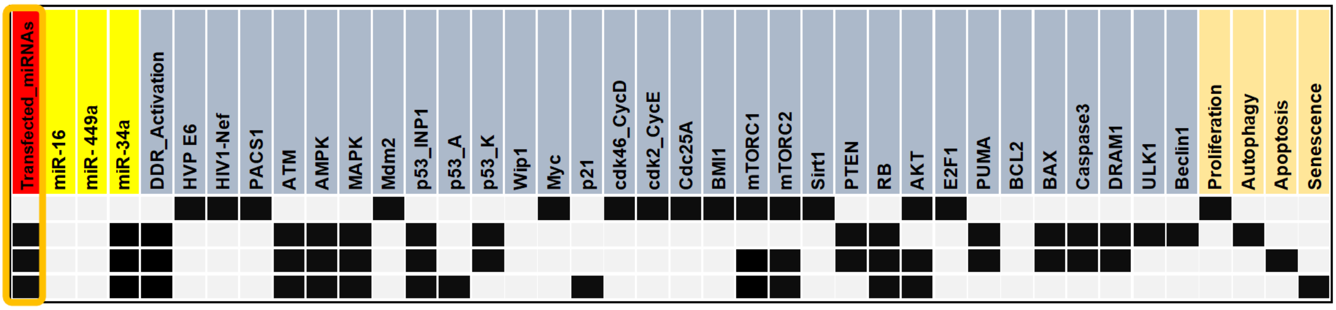

Response/Phenotype: Inhibition of proliferation and induction of senescence, autophagy and apoptosis
